# CRISPR/Cas9 Editing of Starch Branching Enzyme Genes Remodels Starch Structure and Improves Nutritional Quality in Sweet Potato

**DOI:** 10.3390/molecules31213862

**Published:** 2026-10-26

**Authors:** Weijuan Fan, Yanshen Li, Ziyin Xu, Haozhen Nie, Yusen Gao, Jun Yang, Hongxia Wang

**Affiliations:** 1 Shanghai Key Laboratory of Plant Functional Genomics and Resources, Shanghai Chenshan Botanical Garden, Shanghai 201602, China; wjfan@sibs.ac.cn (W.F.); liyanshen24@cemps.ac.cn (Y.L.);; 2 CAS Center for Excellence in Molecular Plant Sciences, Shanghai Institute of Plant Physiology & Ecology, Chinese Academy of Sciences, Shanghai 200032, China; 3 University of Chinese Academy of Sciences, Beijing 100049, China; 4 College of Life Sciences, Shanghai Normal University, Shanghai 200234, China

**Keywords:** CRISPR/Cas9, sweet potato, starch branching enzyme, amylopectin structure, resistant starch, starch digestibility

## Abstract

Starch branching enzymes (SBEs) play a central role in determining amylopectin architecture and starch functionality, yet their contribution to starch nutritional quality and crop performance in sweet potato remains unclear. In this study, CRISPR/Cas9 was used to target *SBEI* and *SBEII* in sweet potato, generating lines with reduced SBE activity. Edited lines showed markedly increased amylose and resistant starch contents compared with the wild type. Amylopectin chain length analysis revealed a decrease in short chains and a relative increase in longer chains, indicating substantial remodeling of branch structure. Microscopic observations further showed reduced birefringence and structural irregularities in starch granules. These structural changes resulted in altered physicochemical properties, including decreased swelling power and solubility, elevated gelatinization temperature, and modified pasting behavior. In vitro digestion assays demonstrated significantly reduced starch digestibility and lower predicted glycemic index values. Whole-genome resequencing confirmed the stable integration of T-DNA and predicted limited insertion-associated effects, further supporting that the improved starch traits were primarily derived from targeted SBE editing. Importantly, storage root yield remained comparable to that of wild-type plants. Collectively, these results demonstrate that CRISPR/Cas9-mediated *SBE* editing effectively improves starch nutritional quality without compromising yield, providing a promising strategy for developing health-oriented sweet potato varieties.

## 1. Introduction

Sweet potato [*Ipomoea batatas* (L.) Lam.] is one of the most important root crops globally, with an annual production exceeding 100 million tons [1,2,3]. It is widely cultivated due to its high adaptability to biotic and abiotic stresses, and its storage roots are rich in starch, which typically accounts for 50–80% of the dry matter and represents an important resource for food, feed, and industrial applications [4]. Starch, the major component of sweet potato roots, consists of two major glucose polymers, amylose and amylopectin, which differ in the degree of polymerization (DP) of glucan chains and in the frequency of branches [5]. The ratio of amylose to amylopectin and the chain length distribution of amylopectin strongly influence starch physicochemical properties such as gelatinization behavior, swelling capacity, and enzymatic digestibility [5,6,7]. Consequently, genetic manipulation of starch biosynthesis enzymes has become an important strategy for improving starch functionality and nutritional quality.

Starch biosynthesis in plants involves a coordinated network of enzymes located in amyloplasts, including ADP-glucose pyrophosphorylase, starch synthases, starch branching enzymes, and debranching enzymes [8,9,10]. Among these enzymes, starch branching enzymes play a central role by introducing α-1,6 glycosidic linkages into glucan chains during amylopectin biosynthesis [11]. In sweet potato, two main isoforms, *SBEI* and *SBEII*, exhibit partially overlapping but distinct substrate preferences. *SBEII* mainly generates short chains, whereas *SBEI* acts preferentially on longer glucan chains [12]. Alteration of SBE activity can therefore substantially modify amylopectin architecture and influence starch properties [13,14].

Starch structure is of considerable technological importance, as it governs key physicochemical behaviors including gelatinization, retrogradation, and crystallization, thereby influencing processing performance, texture, and product stability [15,16]. Moreover, starch structure also plays a crucial role in nutritional quality, as structural variations affect enzymatic digestibility, glycemic index, and resistant starch formation [17,18]. Resistant starch (RS), the fraction of starch that escapes digestion in the small intestine, has attracted considerable attention due to its beneficial health effects [19]. Increased RS intake is associated with improved glycemic control, enhanced gut microbiota fermentation, and reduced risk of metabolic disorders [20]. However, resistant starch levels are generally relatively low in current sweet potato varieties [21]. Therefore, developing sweet potato varieties with increased resistant starch content represents an important goal for improving the nutritional and functional quality of this staple crop and for public health, since sweet potato is a staple crop with increasing demand as a functional food. However, breeding strategies that directly target starch digestibility and glycemic response traits remain limited.

Previous studies have shown that suppression of *SBE* genes can increase amylose content and alter starch properties in several crops [14,22,23]. In sweet potato, RNA interference and antisense approaches have also been used to reduce SBE activity, resulting in altered starch structure and increased amylose levels [24]. Nevertheless, these approaches often produce variable gene silencing effects and may not provide precise control over gene function. In addition, the consequences of *SBE* manipulation for the nutritional and functional properties of sweet potato starch remain insufficiently characterized. Importantly, the relationships between starch structural remodeling, digestibility kinetics, and functional properties have not yet been systematically investigated.

Recent advances in genome editing technologies have provided powerful tools for precise genetic modification in crop species. The CRISPR/Cas9 system enables targeted mutagenesis of specific genes and has been widely applied to improve agronomic traits, stress tolerance, and nutritional quality in plants. Compared with conventional transgenic or gene-silencing approaches, CRISPR/Cas9 allows more accurate and efficient manipulation of gene function and facilitates the generation of allelic variants with different levels of gene activity [25,26,27]. This capability provides an opportunity to investigate dose-dependent effects of key metabolic enzymes on complex traits such as starch structure and digestibility. In sweet potato, CRISPR/Cas9 is particularly attractive because it enables direct testing of gene function in a crop with a complex genetic background, thereby overcoming some of the limitations of earlier suppression-based approaches, such as incomplete and variable gene silencing, off-target effects on homologous gene family members, and unstable or non-heritable phenotypes [28,29]. In contrast, CRISPR/Cas9-mediated genome editing allows targeted and stable mutagenesis, facilitating more reliable functional characterization

Sweet potato is a hexaploid crop (2*n* = 6*x* = 90) with a highly heterozygous and complex genome, posing substantial challenges for genetic analysis and conventional breeding. According to the current reference genome annotation, one *SBEI* locus (g51137) and one *SBEII* locus (g25144) have been identified as key genes involved in starch branching. However, because sweet potato is a hexaploid species, these loci are represented by multiple homeologous alleles distributed across homologous chromosome sets, creating considerable genetic redundancy. This genomic complexity complicates functional characterization using traditional approaches. In this context, CRISPR/Cas9-mediated genome editing provides an effective strategy for simultaneously targeting multiple allelic copies, thereby facilitating functional analysis and precise improvement of starch-related traits in sweet potato. In the present study, CRISPR/Cas9 genome editing was used to target *SBEI* and *SBEII* in sweet potato. Edited lines were comprehensively characterized with respect to starch molecular structure, physicochemical properties, digestibility, and agronomic performance. We found that editing of *SBEI* or *SBEII* reduced starch branching activity, remodeled amylopectin fine structure toward a less-branched architecture, and consequently increased resistant starch content and reduced starch digestibility without compromising storage root yield. The results provide new insights into the role of *SBE* in starch biosynthesis and demonstrate that genome editing is a reliable approach for improving the nutritional quality of sweet potato.

## 2. Results

### 2.1. CRISPR/Cas9 Editing of SBEI and SBEII Reduced Transcript Abundance and SBE Activity

**Figure 1:**
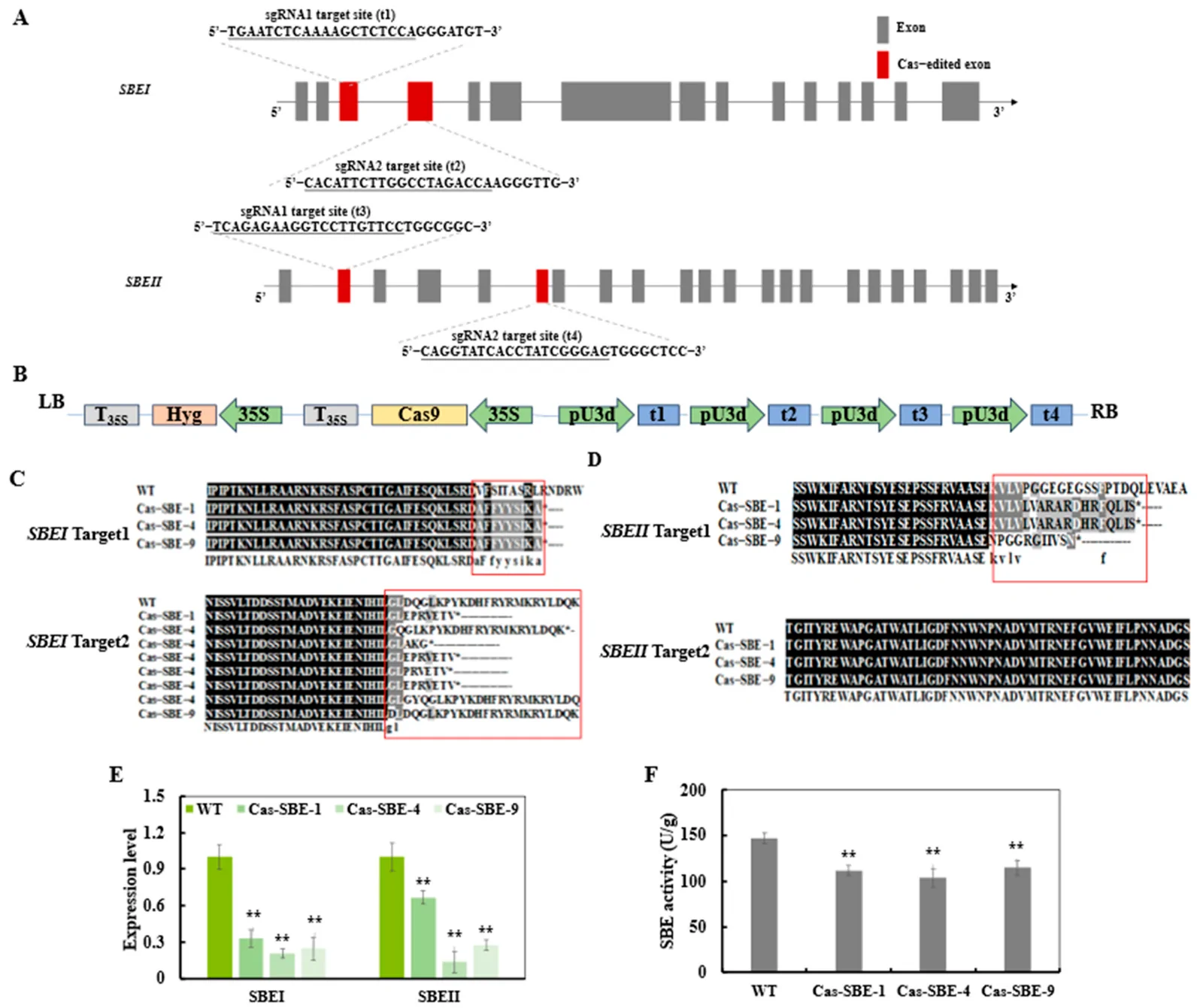
CRISPR/Cas9-mediated editing of *SBEI* and *SBEII* genes in sweet potato and functional characterization of edited lines. (**A**) Schematic diagram of *SBEI* and *SBEII* gene structures and sgRNA target sites. Gray boxes represent exons; red boxes indicate sgRNA target exons. The sequences of four sgRNAs (t1–t4) targeting *SBEI* (t1, t2) and *SBEII* (t3, t4) are shown, with the PAM motifs highlighted in red. (**B**) T-DNA vector construct used for CRISPR/Cas9-mediated gene editing. The construct contains a Cas9 expression cassette driven by the CaMV 35S promoter, and four sgRNA expression cassettes driven by the *Arabidopsis* ubiquitin promoters (pU3d). (**C**,**D**) Translated protein sequences of the target regions of the *SBEI* gene (**C**) and *SBEII* gene (**D**) (target1 & target2) in the WT and independently edited transgenic lines (Cas-SBE-1, Cas-SBE-4, and Cas-SBE-9), illustrating amino acid alterations at the target site. *, premature termination of the protein sequence due to frameshift mutations. red boxes, regions with amino acid alterations (insertions, deletions, or substitutions) caused by CRISPR/Cas editing. (**E**) Relative expression levels of *SBEI* and *SBEII* in WT and edited lines, as determined by qRT-PCR. (**F**) Starch branching enzyme (SBE) activity in WT and edited lines. *p* < 0.01 (**) indicates significant differences compared with WT (Student’s *t*-test).

To dissect the function of starch branching enzymes in sweet potato, we generated transgenic plants expressing sgRNAs targeting *SBEI* and *SBEII* genes using the CRISPR/Cas9 system (Figure 1A,B). Following *Agrobacterium*-mediated transformation, a total of 10 independent transgenic lines were regenerated. To identify edited lines, fragments flanking the sgRNA target sites in *SBEI* and *SBEII* were amplified by PCR, and the amplicons were subjected to next-generation sequencing and analyzed using the High-throughput Tracking of Mutations (Hi-TOM) platform (http://www.hi-tom.net/hi-tom/, accessed on 30 April 2024) [30].

To further characterize the extent and molecular consequences of CRISPR/Cas9-mediated genome editing, mutation profiles at the targeted *IbSBEI* and *IbSBEII* loci were quantitatively analyzed using the Hi-TOM platform. The results revealed diverse mutation patterns among the regenerated edited lines, including insertions, deletions, and nucleotide substitutions (Table S2, Figure S2). Small insertions and deletions (indels) represented the predominant mutation types, with indel sizes ranging from 1 to 35 bp, mainly occurring within the sgRNA target regions adjacent to the PAM sites. The Hi-TOM analysis demonstrated substantial variation in editing efficiency among target loci and independent edited lines (Table S2). Because the amplicons were not phased with homeolog-specific markers, the present Hi-TOM data do not allow the detected mutations to be unambiguously assigned to individual homeologous copies of *SBEI* or *SBEII*.

To further evaluate the functional consequences of these mutations, the deduced protein sequences of the edited alleles were compared with those of the wild type (WT). As shown in Figure 1C,D, translation analysis showed that all Cas-SBE lines carried premature termination mutations at *SBEI* Target1, while at *SBEI* Target2, Cas-SBE-1 and Cas-SBE-4 harbored substitution or deletion mutations leading to premature termination, whereas Cas-SBE-9 showed an SNP without an apparent amino acid change; similarly, all three lines carried deletion-induced premature termination mutations at *SBEII* Target1, whereas no obvious amino acid changes were detected at SBEII Target2. CRISPR/Cas9-induced mutations resulted in diverse amino acid alterations, including substitutions, frameshift mutations, and premature termination codons (indicated by asterisks). Several indel mutations disrupted the reading frame, leading to predicted truncated proteins, whereas other mutations caused amino acid substitutions that could potentially compromise protein function. Consistent with these predicted molecular effects, all edited lines exhibited significantly reduced *SBEI*/*SBEII* transcript abundance and SBE enzyme activity compared with WT plants (Figure 1E,F), confirming that the induced mutations impaired SBE function.

Notably, the extent of genome editing was associated with the magnitude of phenotypic alterations. Among the representative edited lines, Cas-SBE-4 exhibited the highest mutation proportion and a broader mutation spectrum, which corresponded to the strongest reduction in SBE activity and the most pronounced changes in starch structure. Cas-SBE-9 and Cas-SBE-1 showed relatively lower editing levels and corresponding differences in starch-related traits, suggesting a quantitative relationship between editing intensity and functional outcomes.

### 2.2. Whole-Genome Resequencing (WGS) Identified T-DNA Insertion Sites and Evaluated Potential Insertion-Associated Effects

To further characterize the genomic consequences of CRISPR/Cas9 transformation and evaluate whether random T-DNA integration contributed to the observed phenotypic changes, WGS was performed on three representative edited lines (Cas-SBE-1, Cas-SBE-4, and Cas-SBE-9).

Genomic DNA was sequenced using the Illumina NovaSeq platform with paired-end 150 bp reads. Approximately 120 Gb of clean sequencing data were obtained for each sample, corresponding to approximately 40× genome coverage. T-DNA/genome junction reads were identified by alignment against the reference sweet potato genome, allowing for the precise identification and determination of genomic integration sites. Distinct T-DNA insertion patterns were observed among the edited lines (Figure S1). In Cas-SBE-1, one T-DNA insertion event was identified within the genomic region of *g41718*, annotated as a putative ER lumen protein-retaining receptor C28H8.4 homolog. Expression analysis showed that *g41718* transcript abundance was reduced in Cas-SBE-1 compared with WT. In Cas-SBE-4, two independent T-DNA insertion events were detected. One insertion occurred within *g3661*, annotated as an uncharacterized protein LOC102584122 homolog, whereas the other insertion was located in the genomic region adjacent to *g13647*. Transcript analysis revealed that *g3661* expression was increased in Cas-SBE-4, while *g13647* expression was not significantly altered compared with WT. In Cas-SBE-9, three T-DNA insertion events were identified. One insertion occurred within *g25798*, whereas the other two insertions were located in intergenic regions without annotated protein-coding genes within the surrounding genomic intervals. Expression analysis showed that g25798 transcript abundance was not significantly affected in Cas-SBE-9 compared with WT.

Although some insertion-associated genes displayed altered expression, none of the affected genes are involved in known starch biosynthetic pathways. Moreover, independent edited lines carrying different T-DNA insertion patterns consistently exhibited reduced *SBEI*/*SBEII* expression, decreased SBE activity, and similar starch structural modifications, indicating that the major starch-related phenotypes were primarily caused by targeted disruption of *SBEI* and *SBEII* rather than random T-DNA integration events. As expected from the design of the transformation construct, reads matching the Cas9 expression cassette, the hygromycin resistance gene, and vector backbone fragments were detected in all three edited lines; however, these sequences were recovered exclusively as part of the integrated T-DNA at the insertion loci characterized above, and no additional integration events involving Cas9 or vector fragments were identified elsewhere in the genome. Genome-wide comparison between the edited lines and WT also did not reveal additional structural variations or small insertions/deletions beyond the targeted *SBEI/SBEII* mutations and the T-DNA insertion sites described above. Therefore, together with the targeted SBE mutations, the characterized T-DNA insertions (which include the Cas9 cassette and vector backbone fragments) represent the only detectable genome modifications in these lines.

### 2.3. Growth Phenotype and Agronomic Performance of SBE-Edited Plants

**Figure 2:**
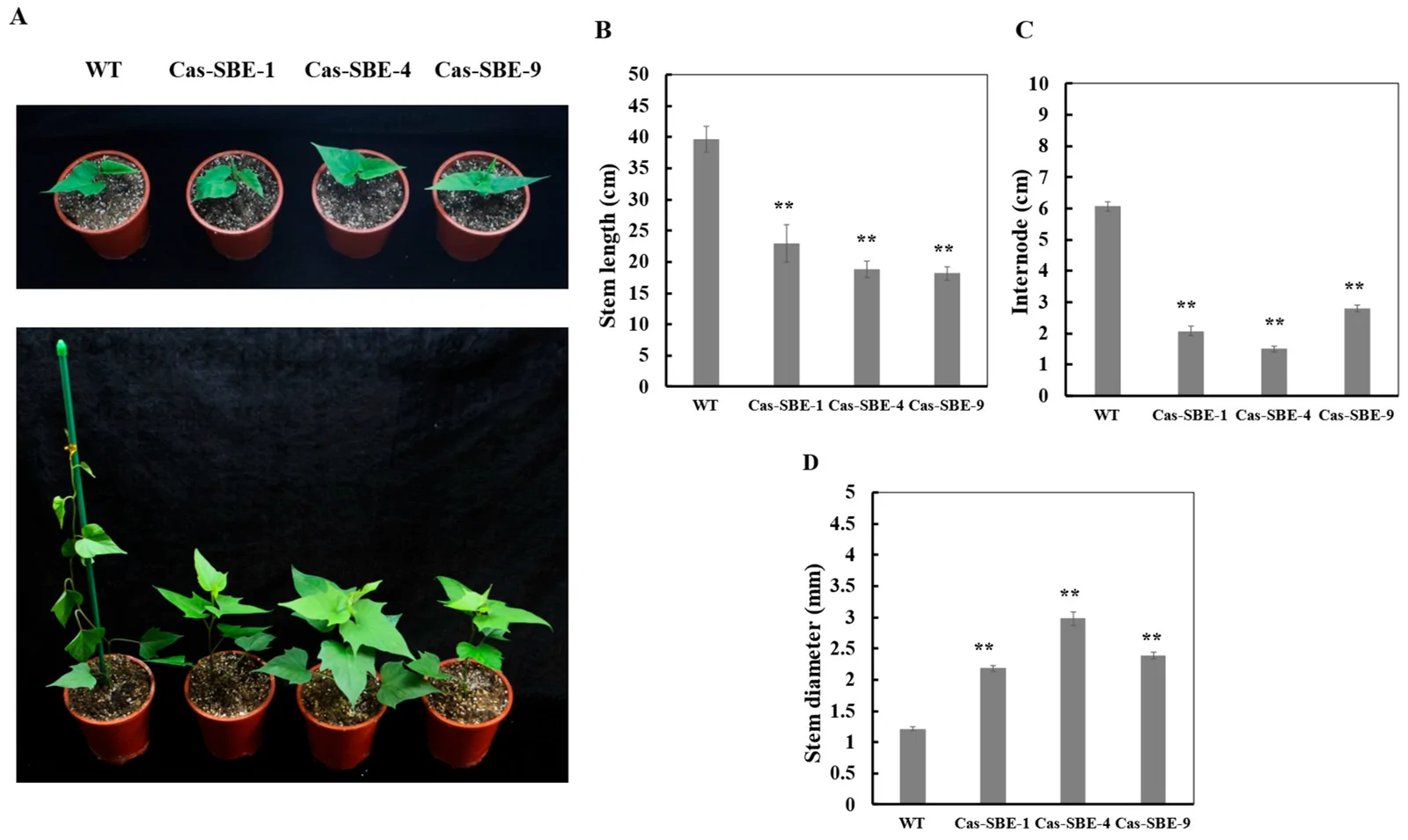
Greenhouse phenotypic analysis of WT and CRISPR/Cas9-edited *SBE* lines. (**A**) Representative photographs of plants at the seedling stage, one week after transfer of tissue culture plantlets to the greenhouse. (top) and after four weeks of growth (bottom). (**B**) Stem length (cm). (**C**) Internode length (cm). (**D**) Stem diameter (mm). Data are presented as the mean ± standard deviation (SD) of 10 independent biological replicates. *p* < 0.01 (**) indicates significant differences compared with WT (Student’s *t*-test).

To evaluate the impact of *SBE* gene editing on plant growth and development, phenotypic traits of WT and the three edited lines were examined under greenhouse conditions. At the seedling stage, all edited lines appeared morphologically similar to WT plants and exhibited no visible developmental defects. However, after four weeks of growth, clear phenotypic differences became evident. WT plants displayed vigorous vertical growth with elongated stems, whereas the edited lines exhibited a more compact architecture characterized by shorter and thicker stems (Figure 2). As shown in Figure 2B, WT plants reached an average stem length of 39.67 ± 2.08 cm, whereas the edited lines showed a significant reduction of 41.5–50.0% compared with WT. Consistent with this reduction, the average internode length was also significantly shorter in all edited lines (Figure 2C). In contrast, stem diameter increased markedly, with values 42–54% greater than those observed in WT plants (Figure 2D).

**Figure 3:**
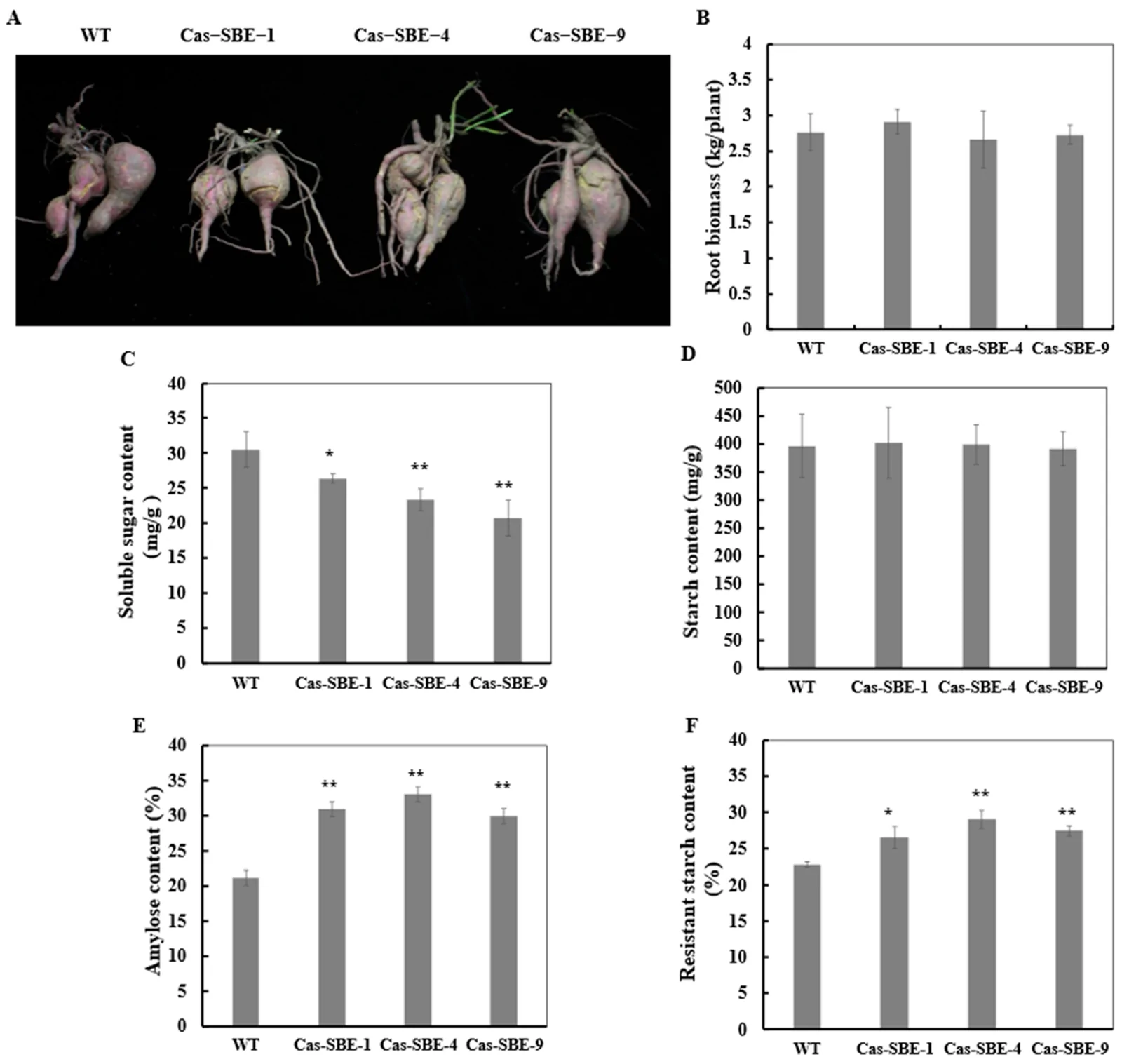
Field trial of CRISPR/Cas9-edited *SBE* lines and biochemical analysis of harvested roots. (**A**) Representative photographs of harvested roots. (**B**) Root biomass of WT and edited lines. Data are presented as the mean ± standard deviation (SD) of 10 independent biological replicates. (**C**) Soluble sugar content. (**D**) Total starch content. (**E**) Amylose content. (**F**) Resistant starch (RS) content. Data are presented as the mean ± standard deviation (SD) of 3 independent biological replicates in (**C**–**F**). *p* < 0.05 (*) and *p* < 0.01 (**) indicate significant differences compared to WT (Student’s *t*-test).

To determine whether these morphological changes affected agronomic performance, WT and edited lines were further evaluated under field conditions for five months (Figure 3). WT and the edited lines produced comparable storage root yields (Figure 3B). No significant differences in yield were detected between the edited lines and WT. Additionally, the number of storage roots per plant remained consistent across genotypes (2–3 storage roots per plant), and no obvious differences in root morphology were observed. These results indicate that *SBE* gene editing alters plant architecture but does not negatively affect storage root yield.

### 2.4. SBE-Edited Storage Roots Accumulate More Amylose and Resistant Starch Without Changing Total Starch Content

To determine whether *SBE* editing affected carbohydrate composition in storage roots, the contents of soluble sugar, total starch, amylose, and resistant starch (RS) were quantified. The soluble sugar content was significantly reduced in all edited lines compared with WT (Figure 3C). In contrast, total starch content remained unchanged in all edited lines (Figure 3D). A substantial increase in amylose content was observed in all edited lines (Figure 3E). Correspondingly, WT starch contained 22.9 ± 1.1% RS, whereas the resistant starch (RS) content in the edited lines was significantly higher than that in WT. (Figure 3F). These results indicate that *SBE* gene editing promotes amylose accumulation and enhances resistant starch formation in sweet potato storage roots.

### 2.5. SBE Editing Remodels Starch Molecular and Granular Structure

#### 2.5.1. Iodine-Binding Properties of Starch from WT and CRISPR/Cas9-Edited SBE Lines

**Figure 4:**
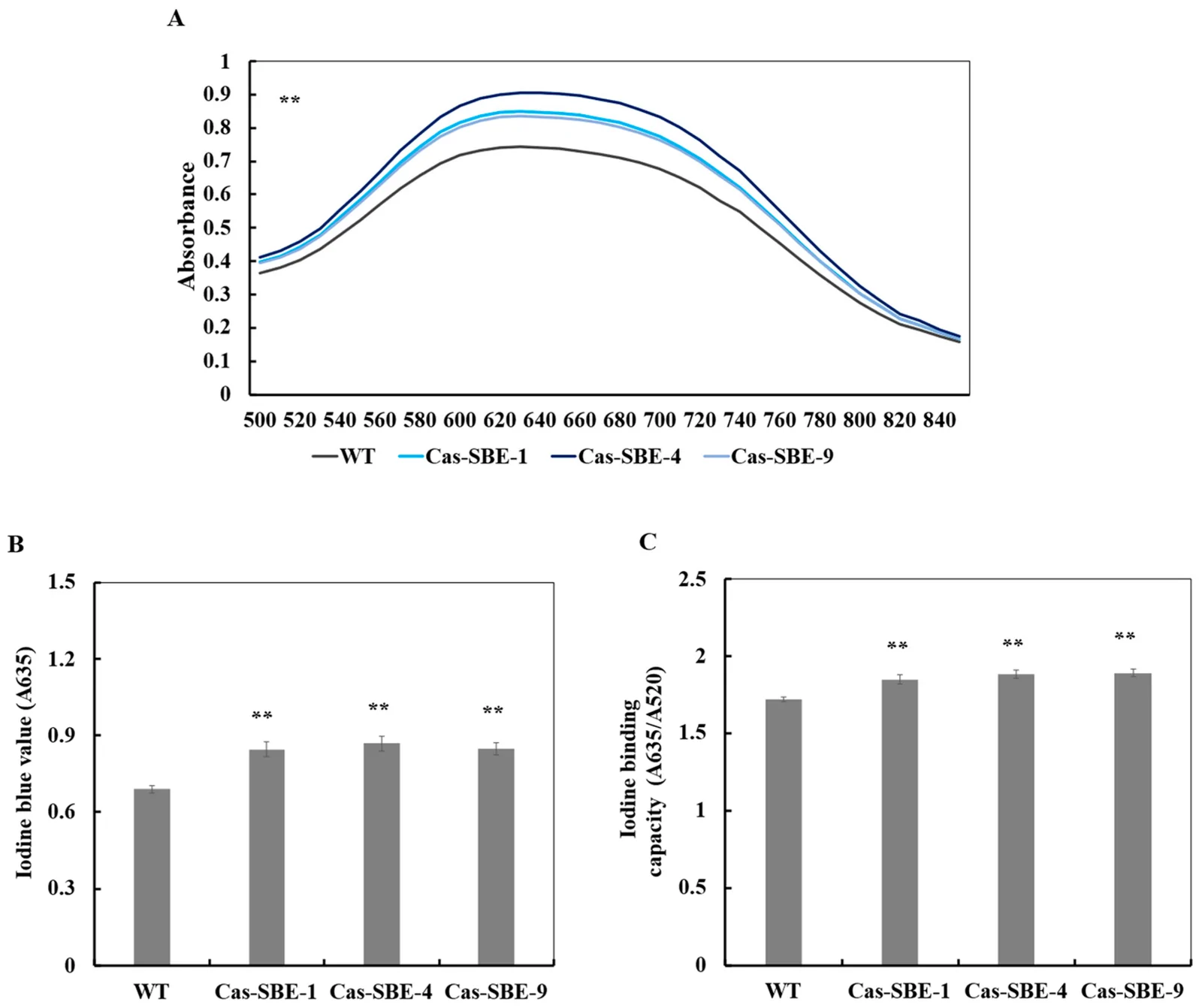
Iodine-binding properties of starch from WT and CRISPR/Cas9-edited *SBE* lines. (**A**) UV-Vis absorption spectra of starch-iodine complexes. (**B**) Iodine blue value (A635, absorbance at 635 nm). (**C**) Iodine-binding capacity (A635/A520). *p* < 0.01 (**) indicates significant differences compared with WT (Student’s *t*-test).

The iodine-binding assay was used to dissect structural differences in starch composition between WT and edited lines. All samples exhibited a characteristic absorption peak at approximately 630 nm, indicating the formation of starch–iodine inclusion complexes (Figure 4A). However, the absorbance intensity at this wavelength was markedly higher in starch from the edited lines than in WT starch. Among the edited lines, Cas-SBE-4 showed the strongest peak, followed by Cas-SBE-9 and Cas-SBE-1. Quantitative analysis using the iodine blue value (A635) further confirmed this trend (Figure 4B). All edited lines exhibited significantly higher values. In addition, the iodine-binding capacity, calculated as the ratio of absorbance at 635 nm to that at 520 nm, was significantly increased in all edited lines (Figure 4C). These results are consistent with the longer glucan chains resulting from reduced SBE activity (Figure 1F).

#### 2.5.2. SBE Editing Shifts Amylopectin Chain Length Distribution Toward Fewer Short Chains and More Long Chains

**Figure 5:**
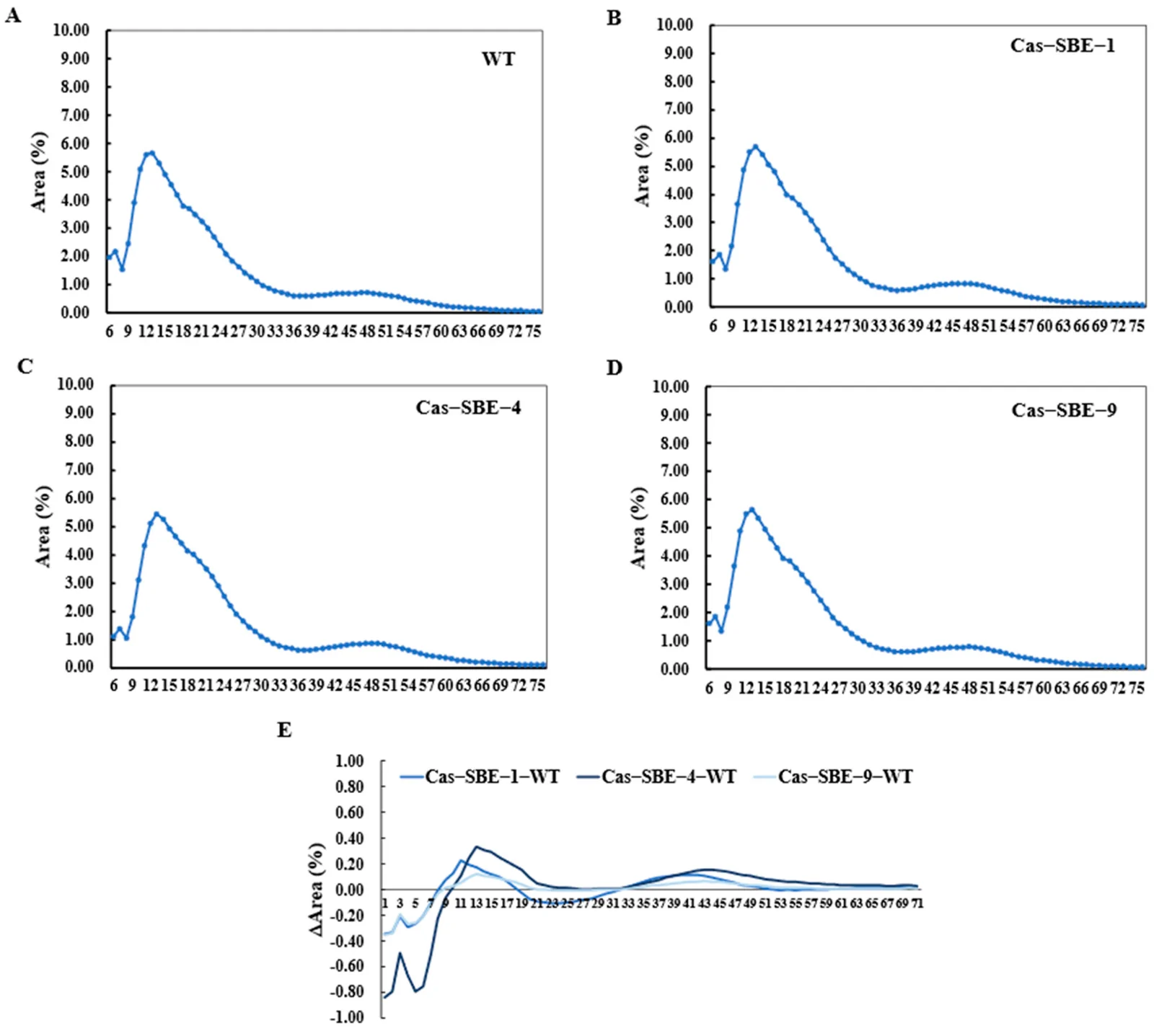
Amylopectin chain length distribution (CLD) of starch from WT and CRISPR/Cas9-edited *SBE* lines. (**A**–**D**) Relative area percentage of amylopectin chains with different degrees of polymerization (DP) in WT (**A**), Cas-SBE-1 (**B**), Cas-SBE-4 (**C**), and Cas-SBE-9 (**D**). (**E**) Differential CLD profile (ΔArea) of edited lines minus WT, where positive value indicates an increase and negative value indicates a decrease in chain length abundance.

The amylopectin chain length distribution of starch from WT and Cas-SBE lines was analyzed by HPAEC–PAD following isoamylase debranching (Figure 5). All samples exhibited similar overall patterns, with a major peak at DP 11–13, a shoulder around DP 18, a minor peak near DP 42–45, and a trough at DP 8, consistent with typical amylopectin structure (Figure 5A–D). Compared with WT, the Cas-SBE lines showed modest but consistent alterations. In the short-chain region (DP < 12), the Cas-SBE lines displayed a slight reduction in very short chains and a relative increase around DP 10–13. In contrast, the proportion of longer chains (DP ≥ 25), particularly in the DP 35–50 range, was slightly elevated in the Cas-SBE lines, with Cas-SBE-4 showing the most pronounced change (Figure 5C). Differential analysis further confirmed a decrease in short chains and a concomitant increase in longer chains in the Cas-SBE lines (Figure 5E). Overall, *SBE* editing resulted in a subtle redistribution of amylopectin chain lengths without altering the general distribution pattern. These data show that CRISPR/Cas9-mediated perturbation of SBE activity does not abolish the overall amylopectin profile, but instead fine-tunes chain-length architecture in a direction consistent with reduced branching.

#### 2.5.3. Starch Granule Morphology

**Figure 6:**
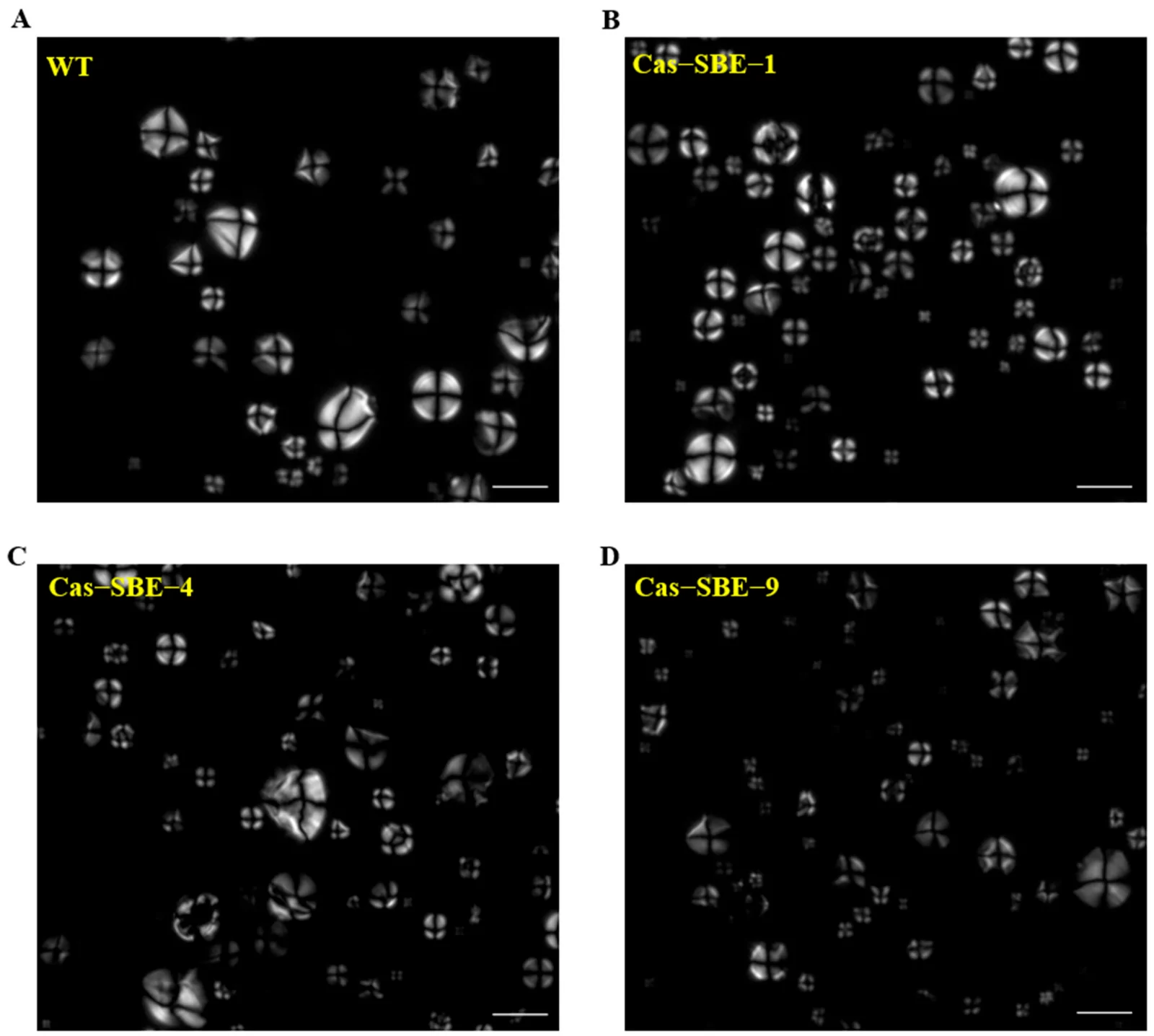
Polarized light microscopy observations of starch granules in WT (**A**) and CRISPR/Cas9-edited *SBE* lines (**B**–**D**). Characteristic Maltese cross birefringence patterns are visible in all samples. The white line in each panel represents the scale bar. Scale bar = 20 μm.

Polarized light microscopy revealed clear differences in starch granule organization between WT and *SBE*-edited lines. WT starch granules were predominantly oval with smooth surfaces and exhibited strong birefringence, displaying typical Maltese cross patterns, indicative of a well-ordered semicrystalline structure (Figure 6A). By contrast, starch granules from Cas-SBE-1, Cas-SBE-4, and Cas-SBE-9 lines showed a noticeable reduction in birefringence intensity (Figure 6B–D). Cas-SBE-1 granules displayed similar birefringence patterns but with a slightly higher proportion of enlarged or irregularly shaped granules (Figure 6B). In contrast, Cas-SBE-4 exhibited the most pronounced structural alterations, including blurred birefringence patterns and frequent morphological irregularities such as fissures and cracks (Figure 6C). Cas-SBE-9 displayed intermediate characteristics (Figure 6D). These structural alterations were more evident in the higher-amylose-content line, Cas-SBE-4, indicating that reduced SBE activity disturbed internal molecular order and thereby altered granule architecture.

**Figure 7:**
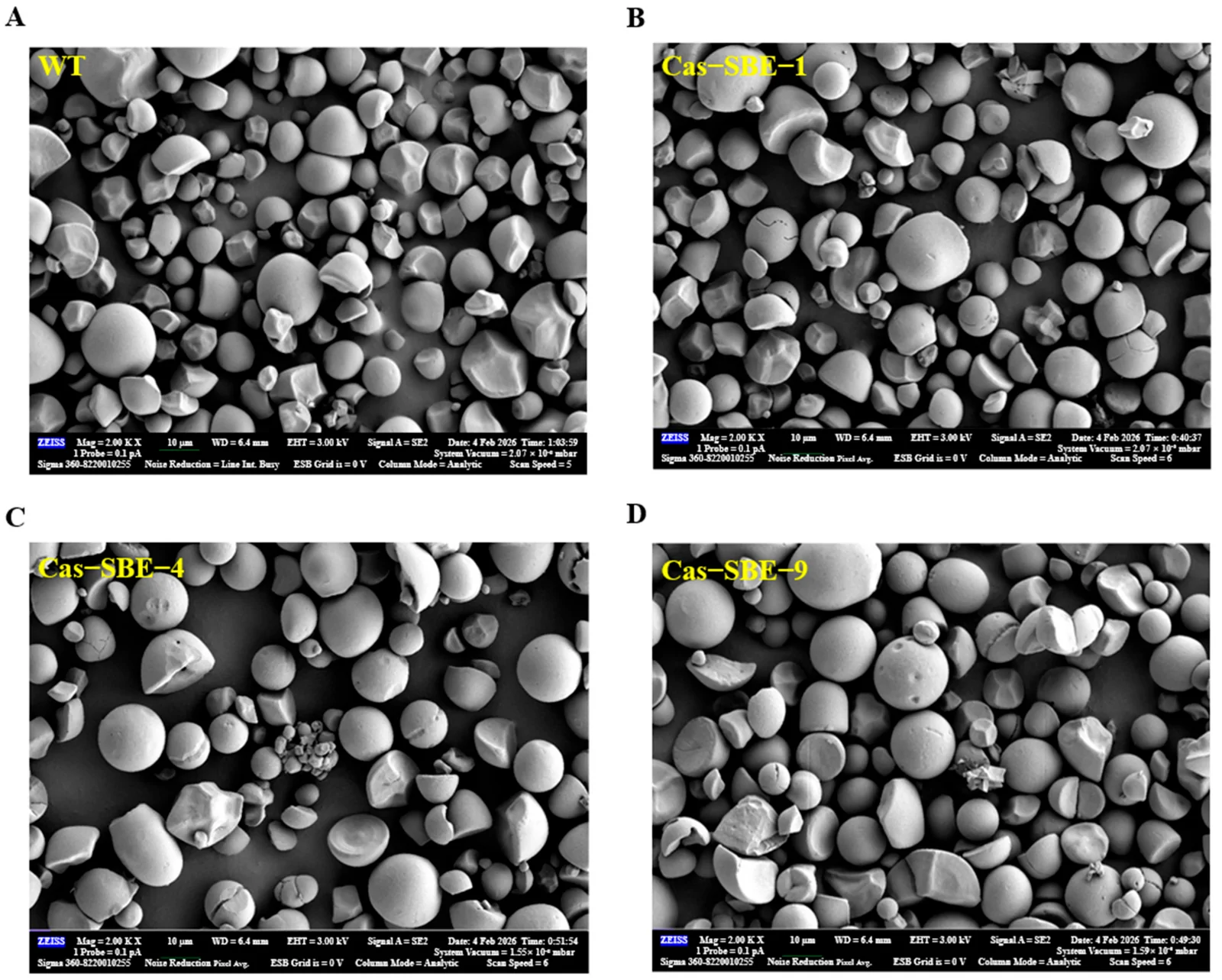
Scanning electron microscopy observations of starch granules from WT (**A**) and CRISPR/Cas9-edited *SBE* lines (**B**–**D**). The green line in each panel represents the scale bar Scale. Scale bar = 10 μm.

Scanning electron microscopy (SEM) analysis did not reveal significant differences in the overall granule shape of starch between the Cas−SBE lines and WT. Various granule shapes, including oval, round, and polygonal forms, were observed in both the Cas−SBE lines and WT (Figure 7). However, obvious structural defects were observed in the Cas-SBE lines. Specifically, starch granules of Cas-SBE-1 were largely similar to those of the WT, but exhibited slight surface indentations and cracks (Figure 7B). Granules of Cas-SBE-4 showed pronounced structural damage, including cracks, fragmentation, and irregular morphology (Figure 7C). In contrast, Cas-SBE-9 displayed moderate surface roughness with occasional indentations, along with sporadic structural abnormalities (Figure 7D). Overall, WT starch granules exhibited smooth surfaces and regular morphology, whereas the *SBE*-edited lines showed varying degrees of surface defects and morphological irregularities. These observations are consistent with previous reports in high-amylose starch, as elevated amylose content can disrupt the semicrystalline structure of starch granules [22,31]. Taken together, the polarized light microscopy and scanning electron microscopy observations indicate that *SBE* editing affects both internal molecular order and surface integrity, with Cas-SBE-4 showing the strongest structural disruption among the lines examined.

### 2.6. Starch Physicochemical Properties

#### 2.6.1. Reduced Swelling Power and Solubility of Starch from Edited Lines

**Figure 8:**
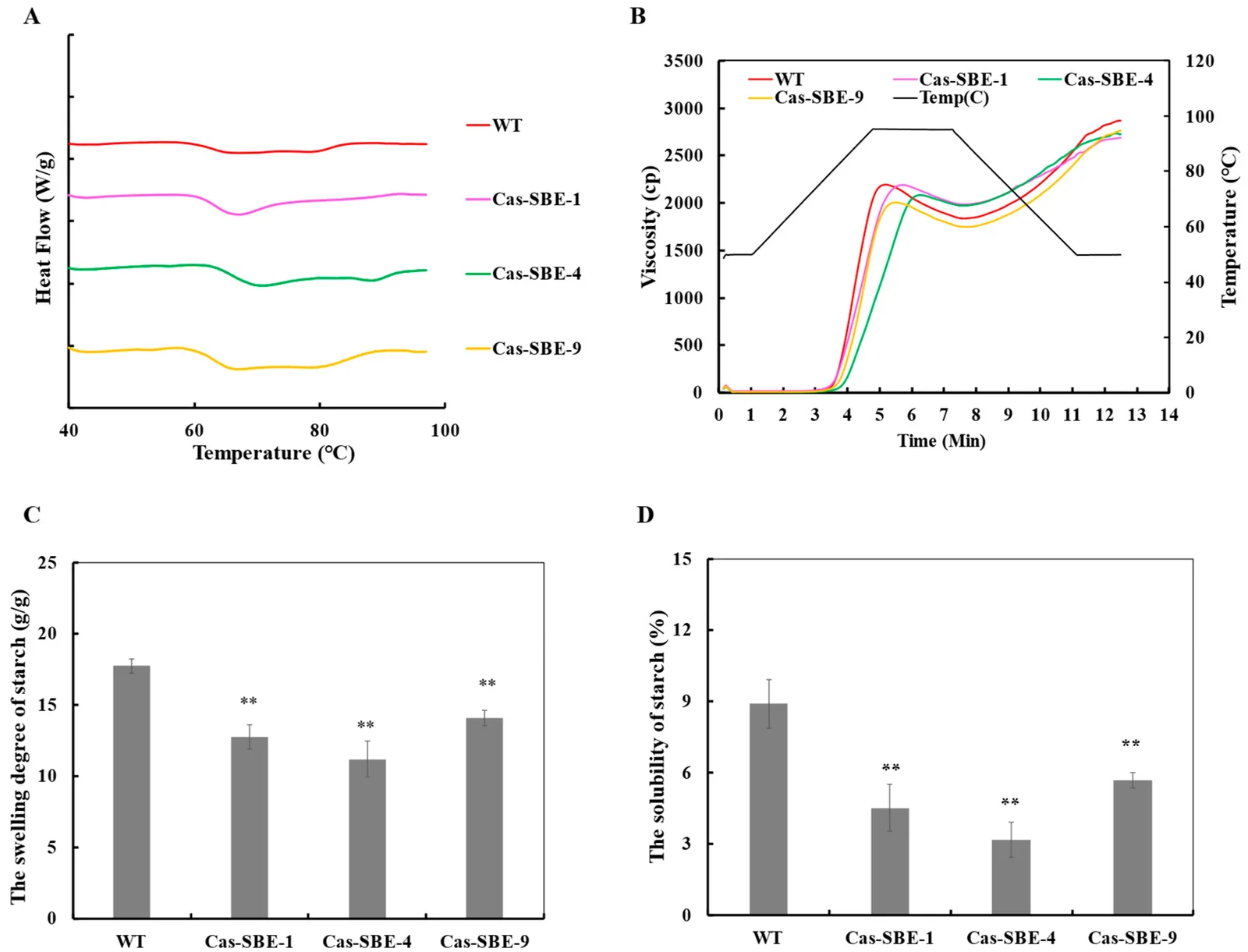
Physicochemical Properties of Starch from WT and CRISPR/Cas9-edited *SBE* lines. (**A**) DSC thermograms showing starch gelatinization endotherms. (**B**) RVA pasting profiles, with viscosity (cP) plotted against time (min) and temperature (°C, black line). (**C**) Swelling degree of starch from WT and edited lines. (**D**) Solubility of starch from WT and edited lines. *p* < 0.01 (**) indicate significant differences compared with WT (Student’s *t*-test).

Swelling power and solubility were measured to evaluate the hydration and structural properties of starch granules. All edited lines exhibited significantly lower swelling power than WT starch (Figure 8C). Similarly, starch solubility was significantly reduced in the edited lines (Figure 8D). The concurrent reduction in swelling power and solubility suggests stronger intermolecular interactions and a more compact internal structure within the starch granules of the edited lines.

#### 2.6.2. Thermal and Pasting Properties of Starch from Edited Lines

Thermal properties of starch were analyzed using differential scanning calorimetry (DSC). All samples exhibited a major endothermic peak corresponding to starch gelatinization (Figure 8A). The complete thermal parameters of all samples, including the onset (To), peak (Tp), and conclusion (Tc) temperatures and the gelatinization enthalpy, are summarized in Table S3. In the edited lines, a small secondary peak (shoulder) was additionally observed on the high-temperature side of the main gelatinization endotherm. The main endotherm reflects the melting of the amylopectin crystalline domains, whereas the secondary transition is most likely attributable to the dissociation of amylose-lipid complexes and/or the melting of more perfect crystallites formed by the enriched long glucan chains, consistent with the increased amylose content of the edited lines. Compared with WT, the gelatinization temperatures of the edited lines increased, while Tp and Tc also increased. In contrast, the gelatinization enthalpy decreased from 12.63 ± 0.38 J/g in WT to as low as 7.26 ± 0.51 J/g in the edited lines (Table S3). These results indicate that starch from the edited lines required higher temperatures to undergo gelatinization but exhibited a lower enthalpy change during the transition.

Consistent with these observations, Rapid Visco Analyzer (RVA) analysis revealed altered pasting properties. The pasting temperature increased in the edited lines, whereas peak viscosity decreased compared with WT starch. Breakdown and setback viscosities were also generally reduced in the edited lines (Figure 8B). These results indicate that *SBE* gene editing significantly modifies the thermal transition and pasting behavior of starch. Together with the swelling and solubility data, these results support the conclusion that molecular remodeling caused by reduced SBE activity is translated into clear changes in starch processing and functional properties.

### 2.7. Starch Digestibility and Predicted Glycemic Index

**Figure 9:**
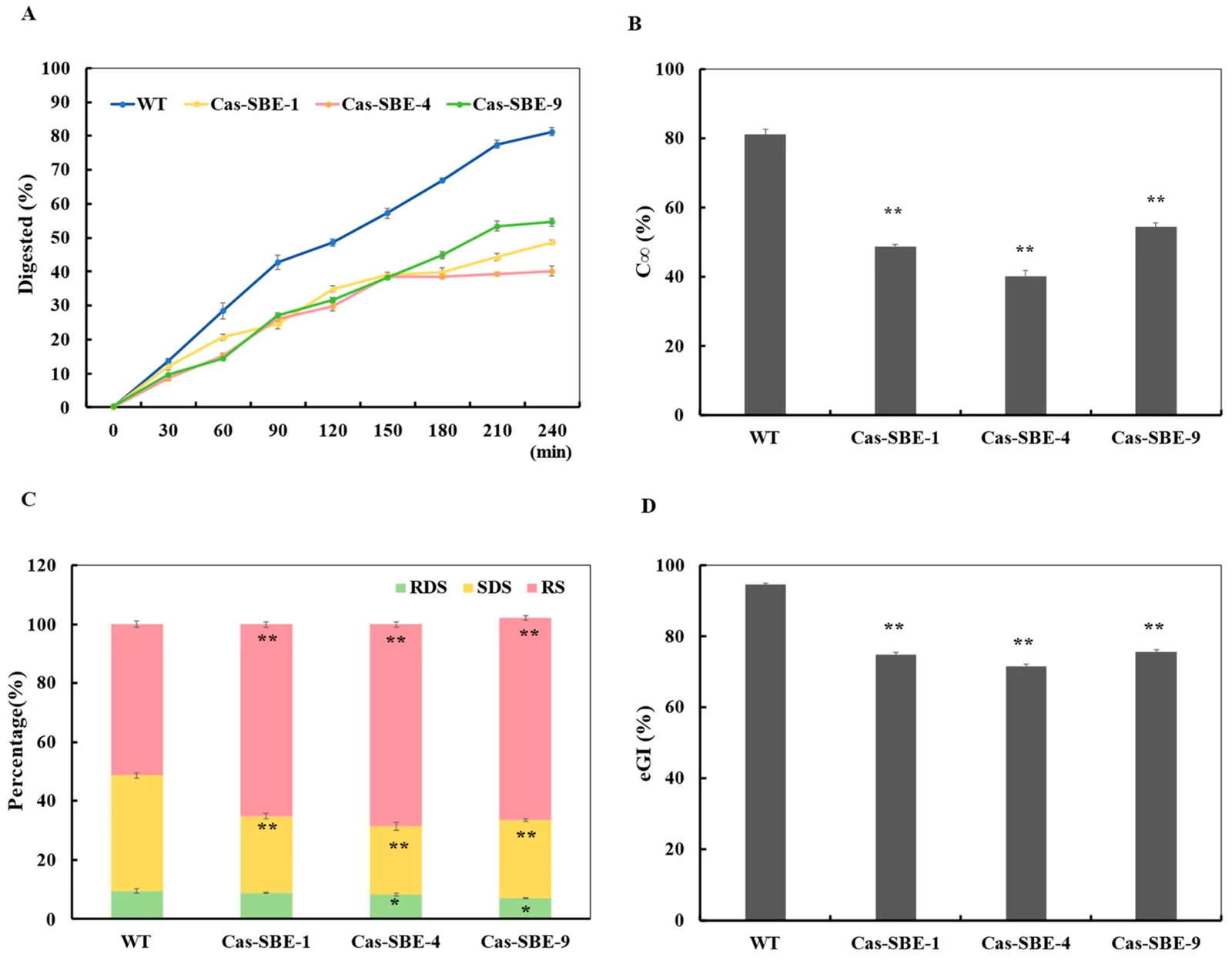
In vitro digestibility and predicted glycemic index of starch from WT and *SBE*-edited lines. (**A**) Time-course hydrolysis curves of starch during in vitro digestion. (**B**) Maximum hydrolysis percentage (C∞) of starch samples. (**C**) Relative proportions of rapidly digestible starch (RDS), slowly digestible starch (SDS), and resistant starch (RS) fractions. (**D**) Predicted glycemic index (eGI) of starch samples. *p* < 0.05 (*) and *p* < 0.01 (**) indicate significant differences compared with WT in (**B**–**D**) (Student’s *t*-test).

The in vitro digestibility of starch was evaluated by monitoring enzymatic hydrolysis over a 240 min digestion period (Figure 9A). Because the starch was extracted and analyzed without a prior cooking or gelatinization step, this assay evaluates the intrinsic digestibility of native starch granules rather than the digestibility of cooked starch. All samples exhibited a biphasic digestion pattern characterized by rapid hydrolysis during the first 90 min followed by a slower digestion phase approaching a plateau. However, starch from the edited lines consistently showed lower hydrolysis rates than WT throughout the digestion process (Figure 9A).

To quantify digestion kinetics, hydrolysis curves were fitted to a first-order kinetic model to obtain the maximum digestible fraction (C∞). The C∞ was substantially lower in the edited lines than in WT (Figure 9B). Furthermore, the distribution of starch fractions was significantly altered. The rapidly digestible starch (RDS) fraction decreased from 9.4% in WT to as low as 6.9% in the Cas-SBE-9 line, whereas the resistant starch (RS) fraction increased markedly from 51.3% in WT to as high as 68.7% in the Cas-SBE-9 line (Figure 9C).

To further evaluate the digestion behavior, the estimated glycemic index (eGI) was also calculated (Figure 9D). WT starch exhibited the highest eGI values, whereas all edited lines showed significantly reduced values, indicating a lower glycemic response. These findings demonstrate that *SBE* gene editing enhances resistance to enzymatic digestion and improves the nutritional properties of starch. Collectively, these results support the central premise that the targeted reduction of branching activity can shift native sweet potato starch toward a slower-digesting, nutritionally improved profile without reducing total starch accumulation or storage root yield.

## 3. Discussion

### 3.1. CRISPR/Cas9-Mediated Mutagenesis of SBE Efficiently Alters Starch Biosynthesis

In this study, CRISPR/Cas9-mediated editing of *SBE* genes resulted in a substantial reduction in both transcript abundance and total SBE enzymatic activity (Figure 1E,F). The edited lines exhibited a 21–29% decrease in SBE activity compared with WT, confirming the successful disruption of starch branching enzyme function. *SBEs* play a central role in amylopectin biosynthesis by introducing α-1,6 glycosidic linkages that generate branch points within the starch molecule [11,32]. Therefore, suppression of SBE activity is expected to alter the balance between short and long glucan chains. Our results are consistent with this expectation and support the interpretation that targeting of *SBEI* and *SBEII* is sufficient to measurably shift starch biosynthesis away from normal branch formation in sweet potato.

Consistent with this role, the edited lines showed markedly increased amylose content and resistant starch levels (Figure 3E,F and Figure 9C). Similar increases in amylose accumulation following suppression of *SBE* genes have been reported in other crops such as rice, maize, and wheat, where reduced branching activity redirects glucan elongation toward more linear structures [23,33,34,35,36]. These results collectively confirm that SBE activity is a key determinant of starch molecular architecture and demonstrate the effectiveness of CRISPR/Cas9 as a tool for targeted modification of starch biosynthesis pathways in sweet potato. Off-target effects and random T-DNA integration remain critical considerations in CRISPR/Cas9-mediated crop improvement, particularly in polyploid species. In this study, WGS (at ~40× coverage) was performed to characterize T-DNA insertion patterns in representative edited lines. Distinct insertion events were identified among Cas-SBE-1, Cas-SBE-4, and Cas-SBE-9. Although some insertion-associated genes, including *g41718* and *g3661*, showed altered transcript abundance, these genes have no known roles in starch biosynthesis. In Cas-SBE-9, one insertion occurred within *g25798*, whereas two additional insertions were located in intergenic regions without annotated neighboring genes. Importantly, independent edited lines carrying different T-DNA insertion patterns exhibited consistent reductions in SBE activity and similar alterations in starch structure and digestibility, indicating that the major metabolic and nutritional phenotypes were primarily caused by targeted disruption of *SBEI* and *SBEII* rather than insertion-associated effects. These results further support the reliability of CRISPR/Cas9-mediated SBE editing for improving sweet potato starch quality. Moreover, although Cas9 and vector backbone sequences were detected, they were confined to the characterized T-DNA insertion loci as integral parts of the integrated T-DNA, and no additional integration events, structural variations, or small indels were identified elsewhere in the genomes of the edited lines, indicating that, apart from the targeted *SBE* mutations, the characterized T-DNA insertions represent the only detectable genome modifications in these materials. More broadly, these findings address an important technology gap in sweet potato improvement by demonstrating that genome editing, together with genomic validation of edited materials, can be used to manipulate a central starch biosynthetic enzyme and generate predictable changes in starch composition.

### 3.2. SBE Editing Remodels Amylopectin Fine Structure

One of the most striking effects of *SBE* editing observed in this study was the alteration of amylopectin chain length distribution. The increased iodine-binding capacity observed in the edited lines supports this structural remodeling (Figure 4). Because iodine preferentially forms inclusion complexes with long glucan chains, the stronger absorbance in the edited lines reflects an increased proportion of long linear segments within the starch polymer.

The edited lines exhibited a moderate reduction in short amylopectin chains (DP <10) accompanied by an slight increase in longer chains (DP > 33) (Figure 5). This shift is consistent with the known biochemical function of SBE enzymes, which normally introduce branch points that generate short glucan chains within the amylopectin molecule [6]. When SBE activity is reduced, fewer branch points are introduced, resulting in the elongation of glucan chains and a relative enrichment of longer chains [37]. Similar changes in chain length distribution have been observed in *SBE*-deficient mutants of cereals and tuber crops, indicating that the mechanism is highly conserved across plant species [23,37,38]. This result provides an initial line of evidence that reduced branching activity changed amylopectin fine structure.

### 3.3. Altered Amylopectin Fine Structure Is Translated into Changes in Starch Granule Architecture

Alterations in molecular structure often translate into changes in starch granule organization. In this study, polarized light microscopy revealed that although the typical Maltese cross birefringence pattern was retained in all samples, the clarity and symmetry of the pattern were partially disrupted in edited lines, particularly Cas-SBE-4 (Figure 6). This suggests that *SBE* editing affects the internal crystalline organization of starch granules. Scanning electron microscopy further revealed morphological alterations on the granule surface, including fissures, rough surfaces, and irregular contours (Figure 7). Such structural defects have also been reported in other high-amylose starches and are often associated with reduced branching and altered crystalline packing within starch granules. These observations indicate that the disruption of SBE activity not only modifies starch molecular structure but also influences granule assembly during starch biosynthesis, leading to detectable changes in granule architecture.

Although the overall amylopectin chain length distribution profile was retained, the consistent redistribution from shorter to longer chains suggests that *SBE* disruption fine-tunes, rather than completely reorganizes, starch granule architecture.

### 3.4. Structural Remodeling of Starch Explains the Altered Physicochemical Properties

A central objective of this study was to determine whether targeted editing of *SBE* genes would lead not only to structural changes in starch, but also to predictable changes in starch functionality. The results strongly support this view. The molecular and morphological changes observed in the edited lines were accompanied by substantial alterations in starch physicochemical properties. The starches from edited lines exhibited significantly reduced swelling power and solubility compared with WT starch (Figure 8C,D). These changes are consistent with the increase in amylose content and enrichment of longer glucan chains, which are expected to strengthen intermolecular associations and limit the ability of starch granules to hydrate and expand. These changes likely reflect stronger intermolecular interactions and a more compact internal structure within the starch granules. Thermal and pasting analyses further demonstrated that *SBE* editing increased gelatinization temperature while reducing gelatinization enthalpy and peak viscosity (Figure 8A,B). Increased gelatinization temperatures indicate that higher thermal energy is required to disrupt crystalline regions within the starch granules. Meanwhile, the reduced peak viscosity and swelling capacity suggest restricted granule expansion during heating. The decrease in gelatinization enthalpy further indicates that the starch granules from edited lines do not simply become more crystalline; rather, their internal organization is qualitatively altered, likely reflecting a different balance between ordered and disordered domains. These findings are consistent with previous studies showing that starches enriched in long glucan chains and amylose generally exhibit reduced swelling and altered pasting behavior. The observed physicochemical changes therefore appear to be direct consequences of the modified amylopectin chain length distribution caused by SBE disruption.

Taken together, these data establish a structure-function relationship in the *SBE*-edited sweet potato lines: reduced branching activity changes glucan length, which alters starch granule architecture, and these changes in turn modify hydration, thermal transition, and pasting behavior.

### 3.5. Reduced Branching Activity Promotes Resistant Starch Formation and Slows Enzymatic Digestion

One of the most significant outcomes of *SBE* gene editing in this study was the pronounced increase in resistant starch (RS in vitro digestion)) content accompanied by a substantial reduction in rapidly digestible starch (RDS). These compositional changes were consistently reflected in the in vitro digestion profiles, where SBE-edited starches exhibited reduced maximum digestibility (C∞) and significantly decreased estimated glycemic index (eGI) compared with WT (Figure 9). Collectively, these results demonstrate that targeted disruption of *SBE* genes effectively shifts starch digestibility toward a slower and more resistant profile, thereby improving its nutritional quality. These observations directly support our hypothesis that editing of *SBEI* and/or *SBEII* would reconfigure starch structure in a manner that reduces enzymatic accessibility and improves nutritional performance.

Mechanistically, the altered digestibility can be attributed to the profound remodeling of starch granule architecture following *SBE* suppression. The edited lines displayed increased amylose content and a higher proportion of long amylopectin chains, both of which are key determinants of digestion resistance. Reduced branching activity limits the formation of short chains while favoring the accumulation of longer glucan chains, which enhances intermolecular associations and promotes the formation of more ordered and densely packed structures. These structural features are known to facilitate double-helix formation and retrogradation, ultimately generating starch fractions that are less accessible to enzymatic hydrolysis. Such a structure–function relationship has been widely documented in both cereal and root crops, indicating that modulation of branching enzyme activity represents a reliable approach for controlling starch digestibility.

Consistent with our findings, spontaneous and induced *SBE* mutants in other crops have demonstrated similar effects on starch structure and nutritional properties. In maize, the classical amylose-extender mutant caused by defects in *SBEIIb* exhibits reduced amylopectin branching, increased amylose content, and elevated resistant starch levels [39]. Likewise, mutations or suppression of *SBEI*, *SBEIIa,* and *SBEIIb* in rice [34] and wheat [23] result in increased proportions of long amylopectin chains, higher amylose accumulation, and reduced starch digestibility. Similar improvements in starch quality have also been achieved through *SBE* suppression or genome editing in potato [22] and cassava [40]. These cross-species comparisons demonstrate that reduced SBE activity consistently promotes the formation of less-branched starch structures with enhanced nutritional properties.

Beyond starch quality improvement, however, the consequences of SBE manipulation on whole-plant architecture and agronomic performance remain poorly understood, particularly in storage-root crops. In the present study, CRISPR-edited sweet potato lines maintained comparable storage-root yield while exhibiting increased RS content, reduced digestibility, and a compact plant architecture characterized by shorter and thicker stems. The occurrence of a compact plant architecture in edited lines, despite different T-DNA insertion backgrounds among independent events, suggests that this phenotype may be associated with altered SBE function or downstream carbon allocation rather than a single insertion-specific effect. However, further genetic dissection will be required to determine whether the architectural changes represent direct pleiotropic consequences of SBE modulation or indirect effects resulting from altered carbohydrate partitioning. Therefore, this study highlights the potential of CRISPR-mediated *SBE* editing as an effective strategy for developing sweet potato varieties with improved nutritional quality without compromising agronomic performance.

Importantly, the distinct kinetic responses observed among different edited lines suggest that *SBE* manipulation influences not only the extent but also the rate of starch digestion. For instance, the reduced C∞ in certain lines indicates a limitation in the proportion of enzymatically digestible starch. This observation underscores the complexity of starch digestion behavior and suggests that fine-tuning SBE activity may allow precise control over both digestion kinetics and nutritional outcomes. This is especially important for crop improvement, as the goal is not solely to increase resistant starch content, but rather to achieve a controlled modulation of starch digestibility.

From a nutritional standpoint, the reduction in eGI values observed in *SBE*-edited lines indicates a lower postprandial glycemic response. Increased RS content is strongly associated with beneficial physiological effects, including improved glycemic control, enhanced insulin sensitivity, and modulation of gut microbiota through fermentation into short-chain fatty acids. Therefore, the coordinated increase in RS and decrease in RDS achieved through *SBE* editing represents a highly desirable modification for the development of functional foods with improved health benefits. Notably, although modifications in starch biosynthesis pathways often raise concerns regarding potential trade-offs with agronomic performance, the edited lines in this study maintained comparable storage root yield in field trials. This finding suggests that targeted editing of *SBE* genes can improve starch nutritional quality without imposing significant penalties on crop productivity, highlighting its feasibility for practical breeding applications.

Sweet potato is an important staple and functional food crop worldwide. Increasing resistant starch content while maintaining yield is therefore a desirable breeding objective. The results of this study demonstrate that CRISPR/Cas9-mediated editing of *SBE* genes provides an effective strategy for improving the nutritional quality of sweet potato starch through targeted modification of starch biosynthesis pathways. Overall, this study demonstrates that *SBE* is a key regulatory node linking starch biosynthesis, starch granule architecture, and digestibility. The integration of genome editing, structural characterization, and nutritional evaluation provides compelling evidence that precise manipulation of starch branching can be harnessed to develop low-glycemic-index crops. These findings not only advance our understanding of starch structure–function relationships but also establish a foundation for the rational design of nutritionally enhanced crops through targeted genome engineering.

Although the present study focused on primary edited plants, several lines of evidence suggest that the observed phenotypic modifications are likely to be stable. Sweet potato is predominantly propagated vegetatively through storage roots or vine cuttings, which minimizes the effects of meiotic segregation and facilitates the maintenance of edited genotypes once established. Here, the edited lines were clonally propagated and evaluated over two consecutive growing seasons; during this period, the major agronomic traits, starch structural characteristics, and digestibility-related parameters exhibited consistent trends. These observations suggest a reasonable degree of phenotypic stability under field conditions.

Furthermore, numerous studies in rice [34], Brassica napus [41], wheat [23], and potato [42] have demonstrated that CRISPR/Cas9-induced mutations are generally stable and heritable across generations. Therefore, the edited alleles identified in this study are expected to remain stable during subsequent vegetative propagation. Nevertheless, because the present work did not directly assess the long-term genetic stability of individual edited alleles, further investigations involving multiple propagation cycles and diverse environments will be necessary to fully evaluate the durability of the edited genotypes and their associated phenotypes. Such studies will also help confirm the absence of unintended genetic or phenotypic changes over time.

From a regulatory perspective, the commercialization prospects of the edited lines depend on their final genetic composition. In China, the Guidelines for the Safety Evaluation of Gene-Edited Plants (Trial) issued in 2022 provide a simplified safety assessment pathway for gene-edited plants that do not contain foreign gene components [43]. In the United States, plants carrying only small insertions or deletions that could also arise through conventional mutagenesis or that lack introduced DNA are exempt from USDA APHIS regulation under the SECURE rule [43]. In the European Union, genome-edited organisms are currently regulated as GMOs following the 2018 ruling of the Court of Justice, although the proposed regulation on new genomic techniques would relax requirements for NGT-1 plants that contain no foreign DNA [43]. The edited lines characterized in the present study still contain the Cas9 expression cassette and the hygromycin resistance gene within the integrated T-DNA, and would therefore be treated as transgenic plants under the current regulations of all three jurisdictions. Because sweet potato is a hexaploid, highly heterozygous and self-incompatible crop, eliminating the T-DNA through sexual segregation is more demanding than in diploid crops: the edited lines must first be induced to flower and crossed with a non-transgenic partner, and the T-DNA insertions and the Cas9-induced *SBEI*/*SBEII* alleles then segregate independently, so large progeny populations and individual genotyping are required to recover plants that have lost the T-DNA while retaining the desired mutant alleles at all six homoeologous copies; in addition, recombination reshuffles the highly heterozygous background of the original line, and several cycles of evaluation would be needed to restore the target phenotype. Nevertheless, once such a plant is identified, it can be fixed and multiplied indefinitely through vegetative propagation.

## 4. Materials and Methods

### 4.1. Plant Materials and Growth Conditions

The wild-type (WT) sweet potato cultivar Xushu 22, a widely cultivated starch-type variety in China, was used as the genetic background for gene editing. In vitro shoot cultures were subcultured on BBM medium (MS salts including vitamins + 30 g/L sucrose, pH5.8) in plant growth chambers under a 16 h photoperiod provided by cool-white fluorescent tubes (50 µmol/m^2^/s), at 25 °C and 50% relative humidity. For greenhouse experiments, plants were grown in pots (15 cm × 15 cm) filled with soil mixture (soil:peat:perlite = 1:1:1 in volume), maintained at 25 ± 2 °C with a 16 h light/8 h dark photoperiod and 50% relative humidity. For the field trial, the WT and the edited lines were planted in the research farm of the CAS Center of Excellence in Molecular Plant Science in Shanghai. And storage roots were harvested at 5 months after planting.

### 4.2. CRISPR/Cas9 Vector Construction and Genetic Transformation

The CRISPR/Cas9 vector was designed to target conserved regions of *SBEI* (GenBank accession no. AB194724) and *SBEII* (GenBank accession no. AB071286.1). Two specific sgRNAs were designed to target the third and fourth exons in sweet potato *SBEI* gDNA, while two specific sgRNAs were designed to target the second and sixth exons in sweet potato *SBEII* gDNA (Figure 1A). *SBEI* sgRNA1 (5′-TGAATCTCAAAAGCTCTCCA- 3′) and sgRNA2 (5′-CACATTCTTGGCCTAGACCA-3′), while *SBEII* sgRNA1 (5′-TCAGAGAAGGTCCTTGTTCC- 3′) and sgRNA2 (5′-CAGGTATCACCTATCGGGAG-3′) were all driven by U3d promoter from *Arabidopsis*. Then, all the fragments were cloned into the pYLCRISPR/Cas9Pubi-H vector [26]. The recombinant vector was introduced into *Agrobacterium tumefaciens* strain LBA4404 via freeze-thaw transformation.

Sweet potato transformation was performed using embryogenic suspension cultures as described previously [44]. Briefly, embryogenic calli were co-cultivated with *A. tumefaciens* for 3 days, then transferred to selection medium containing 10 mg/L hygromycin for 4–6 weeks. Resistant calli were induced to form somatic embryos and regenerated into plantlets. Transgenic lines were confirmed by PCR amplification of the hygromycin resistance gene (HPT) and sequencing of the target *SBE* regions.

### 4.3. Genotyping of CRISPR/Cas9-Edited Lines via Hi-TOM Platform

Genomic DNA was extracted from leaves of CRISPR/Cas9-edited lines using the CTAB method. Approximately 300 bp fragments flanking the *SBE* target sites were amplified with PrimeSTAR Max DNA Polymerase (TaKaRa, Shiga, Japan, Code No. R045Q) using site-specific primers (Table S1). Genotyping was performed using the Hi-TOM platform as described by Liu et al. [30]. Briefly, PCR amplicons were tagged, subjected to high-throughput sequencing, and analyzed to quantify mutation ratios at target sites, enabling rapid and accurate genotyping of edited lines.

### 4.4. WGS and Identification of T-DNA Insertion Sites

To characterize the genomic integration patterns of the CRISPR/Cas9 transformation cassette and evaluate potential insertion-associated effects in the edited lines, whole-genome sequencing (WGS) was performed for three representative edited lines (Cas-SBE-1, Cas-SBE-4, and Cas-SBE-9) and the corresponding wild-type (WT) control. Genomic DNA was extracted from young leaves using the cetyltrimethylammonium bromide (CTAB) method. DNA quality and concentration were assessed using a NanoDrop spectrophotometer and agarose gel electrophoresis. High-quality genomic DNA samples were used for library construction and subjected to paired-end sequencing on the Illumina NovaSeq platform. Approximately 120 Gb of clean sequencing data were generated for each sample, providing an average genome coverage depth of approximately 40×. Raw sequencing reads were filtered to remove low-quality reads, adapter sequences, and potential contaminant sequences. Clean reads were aligned against the reference sweet potato genome using Minimap2 software, version 2.31 (https://github.com/lh3/minimap2, accessed on 5 August 2026) [45]. Reads containing sequences derived from the CRISPR/Cas9 T-DNA cassette were identified by mapping against the transformation vector sequence. The junction reads spanning both T-DNA and genomic sequences were extracted to determine the genomic integration sites.

The genomic regions flanking the identified T-DNA insertion sites were annotated based on the sweet potato reference genome. Genes located within or adjacent to the insertion regions were identified, and their functional annotations were obtained from public genome databases. To evaluate potential transcriptional effects caused by T-DNA integration, the expression levels of insertion-associated genes were analyzed using qRT-PCR. The expression patterns of these genes were compared between WT and edited lines to determine whether T-DNA insertion resulted in detectable changes in neighboring gene expression.

### 4.5. Analysis of SBE Gene Expression and Enzyme Activity

Total RNA was extracted from sweet potato storage root using the Trizol reagent (TIANGEN, Beijing, China) and reverse-transcribed into cDNA using the PrimeScript RT Reagent Kit (TOYOBO, Osaka, Japan). Quantitative real-time PCR (qRT-PCR) was performed on a CFX96 Real-Time PCR System (Bio-Rad, Hercules, CA, USA) using gene-specific primers (Table S1). The *Actin* gene was used as an internal reference, and relative expression levels were calculated using the 2^−ΔΔCt^ method [46].

SBE enzyme activity was measured using the Starch Branching Enzyme Activity Assay Kit (BC1865, Beijing Solarbio Science & Technology Co., Ltd., Beijing, China) according to the manufacturer’s instructions. Briefly, storage root tissue was homogenized in extraction buffer, centrifuged at 12,000× *g* for 10 min, and the supernatant was used as the crude enzyme extract. The reaction mixture contained enzyme extract, substrate (amylose), and reaction buffer, and absorbance was measured at 540 nm after incubation at 37 °C for 30 min.

### 4.6. Carbohydrate Composition Analysis of Storage Roots

#### 4.6.1. Determination of Soluble Sugar and Total Starch

Storage roots of WT and edited lines were harvested, ground into powder, and dried at 80 °C for approximately 48 h to a constant dry weight (DW). The dried samples (30 mg) were used for the determination of soluble sugar and total starch contents. Soluble sugar and total starch were measured using the Plant Soluble Sugar Content Assay Kit (BC0030, Beijing Solarbio Science & Technology Co., Ltd., Beijing, China) and the Starch Content Assay Kit (BC0700, Beijing Solarbio Science & Technology Co., Ltd., Beijing, China), respectively, according to the manufacturer’s instructions. Briefly, soluble sugars were extracted from the dried powder with distilled water in a boiling water bath, whereas for total starch, soluble sugars were first removed by extraction with 80% ethanol at 80 °C for 30 min, and the retained starch was gelatinized in a boiling water bath and hydrolyzed to glucose. The released glucose in both assays was quantified by the anthrone–sulfuric acid colorimetric method at 620 nm against a glucose standard curve; starch content was calculated from the glucose equivalent using a conversion factor of 1.11, and soluble sugar content was expressed as mg glucose equivalents per g DW.

#### 4.6.2. Starch Extraction and Purification

Starch was extracted from mature sweet potato storage roots as described previously [31]. Storage roots were peeled, cut into small pieces, and homogenized in distilled water. The homogenate was filtered through four layers of cheesecloth. The precipitate was resuspended in water, and washed with distilled water several times, and dried at 40 °C for 24 h. The dried starch was ground into a fine powder and passed through a 100-mesh sieve for subsequent analysis.

#### 4.6.3. Resistant Starch Contents

RS content was determined using the Resistant Starch Assay Kit (BC8520, Beijing Solarbio Science & Technology Co., Ltd., Beijing, China). Samples were incubated with pancreatic α-amylase and amyloglucosidase (AMG) in a shaking water bath at 37 °C for 16 h to solubilize and hydrolyze the non-resistant starch. The reaction was terminated with an equal volume of ethanol, and after centrifugation, the RS pellet was washed twice with 50% (*v*/*v*) ethanol, dissolved in 2 M KOH with vigorous stirring, and neutralized with sodium acetate buffer. The dissolved RS was quantitatively hydrolyzed to D-glucose with AMG, and glucose was measured using the glucose oxidase/peroxidase (GOPOD) reagent at 510 nm against a D-glucose standard.

#### 4.6.4. Amylose Content

Amylose content was determined using the colorimetric method. Starch samples (10 mg) were dissolved in 1 mL of 95% ethanol and 9 mL of 1 M NaOH, boiled for 10 min, and cooled to room temperature. A 1 mL aliquot was mixed with 40 mL of distilled water and 1 mL of 1 M HCl, then 2 mL of iodine reagent (0.2% I_2_ and 2% KI) was added. Absorbance was measured at 620 nm, and amylose content was calculated using a standard curve generated with pure amylose and amylopectin (A0512, 10120, Sigma, St. Louis, MO, USA). This assay estimates apparent amylose content using a calibrated iodine color response after alkaline dissolution.

### 4.7. Determination of Starch Structural Parameters

#### 4.7.1. Chain Length Distribution (CLD)

Amylopectin CLD was measured as previously described [31]. First, the isolated sweet potato starches were enzymatically debranched for quantification of chain-length distributions. In brief, 5 mg of sweet potato starch sample was digested with isoamylase enzyme (15284, Sigma, St. Louis, MO, USA). The debranched starch samples were then used to quantify the amylopectin using high-performance anion-exchange chromatography with pulsed amperometric detection (ICS5000+, Thermo Fisher Scientific, Waltham, MA, USA).

#### 4.7.2. Iodine-Binding Capacity

Iodine-binding capacity was analyzed by UV-Vis spectroscopy. Starch samples (2 mg) were dissolved in 20 mL of distilled water, boiled for 10 min, and cooled to 25 °C. Iodine reagent (0.1 M I_2_-KI) was added to a final concentration of 0.001 M, and the mixture was incubated for 15 min. Absorbance was measured from 500 to 850 nm, and the iodine blue value was defined as the absorbance at 635 nm. Iodine-binding capacity was calculated as the ratio of absorbance at 635 nm (amylose–iodine complex) to that at 520 nm (amylopectin-iodine complex), which characterizes the broader iodine-binding spectral response and provides an iodine-binding index.

#### 4.7.3. Granule Morphology and Crystalline Structure

Scanning electron microscopy (Carl Zeiss AG, Oberkochen, Germany) was used to observe starch granule morphology. Starch samples were sprinkled onto double-sided tape, sputter-coated with gold, and imaged at an accelerating voltage of 5 kV.

Polarized light microscopy (LSM 880NLO, Carl Zeiss AG, Oberkochen, Germany) was used to analyze crystalline structure. Starch samples were suspended in water on a glass slide, covered with a coverslip, and observed under polarized light. Maltese cross patterns were recorded at 400× magnification.

### 4.8. Physicochemical Properties of Starch

#### 4.8.1. Swelling Power and Solubility

Swelling power and solubility were determined as described previously. Starch samples (0.5 g) were mixed with 25 mL of distilled water, incubated in a water bath at 95 °C for 30 min, and centrifuged at 3000× *g* for 10 min. Swelling power was calculated as the ratio of the wet sediment weight to the dry starch weight. Solubility was calculated as the percentage of dry matter in the supernatant relative to the initial dry starch weight.

#### 4.8.2. Pasting Properties

Pasting properties were analyzed using a Rapid Visco-Analyzer (RVA−4C; Newport Scientific Pty. Ltd., Warriewood, Australia). Starch samples (3 g, dry basis) were mixed with 25 mL of distilled water, and the slurry was heated from 50 °C to 95 °C at a rate of 12 °C/min, held at 95 °C for 5 min, cooled to 50 °C at 12 °C/min, and held at 50 °C for 2 min.

#### 4.8.3. Thermal Properties

The starch thermal properties were analyzed by a differential scanning calorimeter (DSC Q2000). 3 mg starch was added to 9 µL distilled water in an aluminum pan and incubated at room temperature for 24 h. With an empty aluminum pan as a reference, the sample was heated at a speed of 10 °C/min from 30 to 105 °C, and the gelatinization temperature and enthalpy were determined and recorded (Universal Analysis 2000, TA Instruments Ltd., Crawley, UK).

### 4.9. In Vitro Starch Digestibility Analysis

In vitro starch digestibility was determined using the method described previously [43]. All digestibility analyses were performed on raw (native, ungelatinized) starch. Starch samples (100 mg) were mixed with 10 mL of simulated gastric fluid (pH 2.0) and incubated at 37 °C for 30 min. The pH was adjusted to 6.9 with 0.2 M sodium phosphate buffer, and 5 mL of enzyme mixture (α-amylase and amyloglucosidase, A3176 and A7095, Sigma, St. Louis, MO, USA) was added. Aliquots (0.5 mL) were taken at 0, 5, 10, 15, 20, 30, 60, 90, 120, 150, 180, 210, and 240 min, and the reaction was terminated by boiling for 10 min. Glucose content was measured using the Glucose Assay Kit (BC2505, Beijing Solarbio Science & Technology Co., Ltd., Beijing, China, China), and rapidly digestible starch (RDS), slowly digestible starch (SDS), and resistant starch (RS) were calculated as follows:

RDS (%) = (Glucose at 20 min × 0.9)/Total starch × 100

SDS (%) = (Glucose at 120 min − Glucose at 20 min) × 0.9/Total starch × 100

RS (%) = (Total starch − RDS − SDS)/Total starch × 100

The starch hydrolysis kinetics were further analyzed using a first-order kinetic model:

*C_t_* = *C*_∞_(1 − *e*^−*kt*^)

where *C_t_* is the percentage of starch hydrolyzed at time *t*, *C*_∞_ is the maximum hydrolysis percentage, and *k* is the kinetic constant. The parameters *C*_∞_ and *k* were estimated by non-linear regression using experimental hydrolysis data.

The hydrolysis index (HI) was calculated as the ratio of the area under the hydrolysis curve (AUC) of the sample to that of the reference (white bread) over 0–240 min:

$$\mathrm{HI}=\frac{\mathrm{AU}C_{\mathrm{sample}}}{\mathrm{AU}C_{\mathrm{reference}}}\times 100$$

where AUC was calculated using the trapezoidal rule.

The estimated glycemic index (eGI) was calculated according to the equation proposed by Goñi et al.:

*eGI* = 39.71 + 0.549 × *HI*

### 4.10. Statistical Analysis

All experiments were performed with three independent biological replicates, unless otherwise stated, and data are presented as mean ± standard deviation (SD). Significant differences between WT and edited lines were determined using Student’s *t*-test, with *p* < 0.05 and *p* < 0.01 considered statistically significant.

## 5. Conclusions

CRISPR/Cas9-mediated editing of *SBEI* and *SBEII* effectively remodeled starch granule architecture in sweet potato. The edited lines exhibited increased amylose and resistant starch contents, altered amylopectin chain length distribution, and improved nutritional properties characterized by reduced digestibility and lower predicted glycemic index.

Importantly, these modifications were achieved without compromising storage root yield. These findings highlight the potential of genome editing as a powerful strategy for improving starch quality and developing nutritionally enhanced sweet potato cultivars.

## Data Availability

The data presented in this study are available on request from the corresponding author.

## Supplementary Materials

The following supporting information can be downloaded at: https://www.mdpi.com/article/10.3390/molecules31213862/s1, Figure S1: Identification of T−DNA insertion sites in CRISPR/Cas9−edited sweet potato lines; Figure S2: Sequence alignments of the four sgRNA target regions in SBEI and SBEII in wild−type and CRISPR/Cas9−edited sweet potato lines; Table S1: List of the primers used in this study; Table S2: Hi−TOM quantification of mutation spectra at the four sgRNA target sites in the Cas−SBE line; Table S3: Thermal properties of SBE−mutated sweet potato determined by a differential scanning calorimeter.
