# Supplementary material for: CRISPR/Cas9 Editing of Starch Branching Enzyme Genes Remodels Starch Structure and Improves Nutritional Quality in Sweet Potato
Source: Molecules. 2026 Oct 26;31(21):3862. doi: 10.3390/molecules31213862 (PMC13765049; doi:10.3390/molecules31213862)
Supplement: Supplementary file 1 [file molecules-31-03862-s001.zip › molecules-4547992-supplementary.pdf]

CRISPR/Cas9 Editing of Starch Branching Enzyme Genes Remodels Starch Structure and Improves Nutritional Quality in Sweet Potato

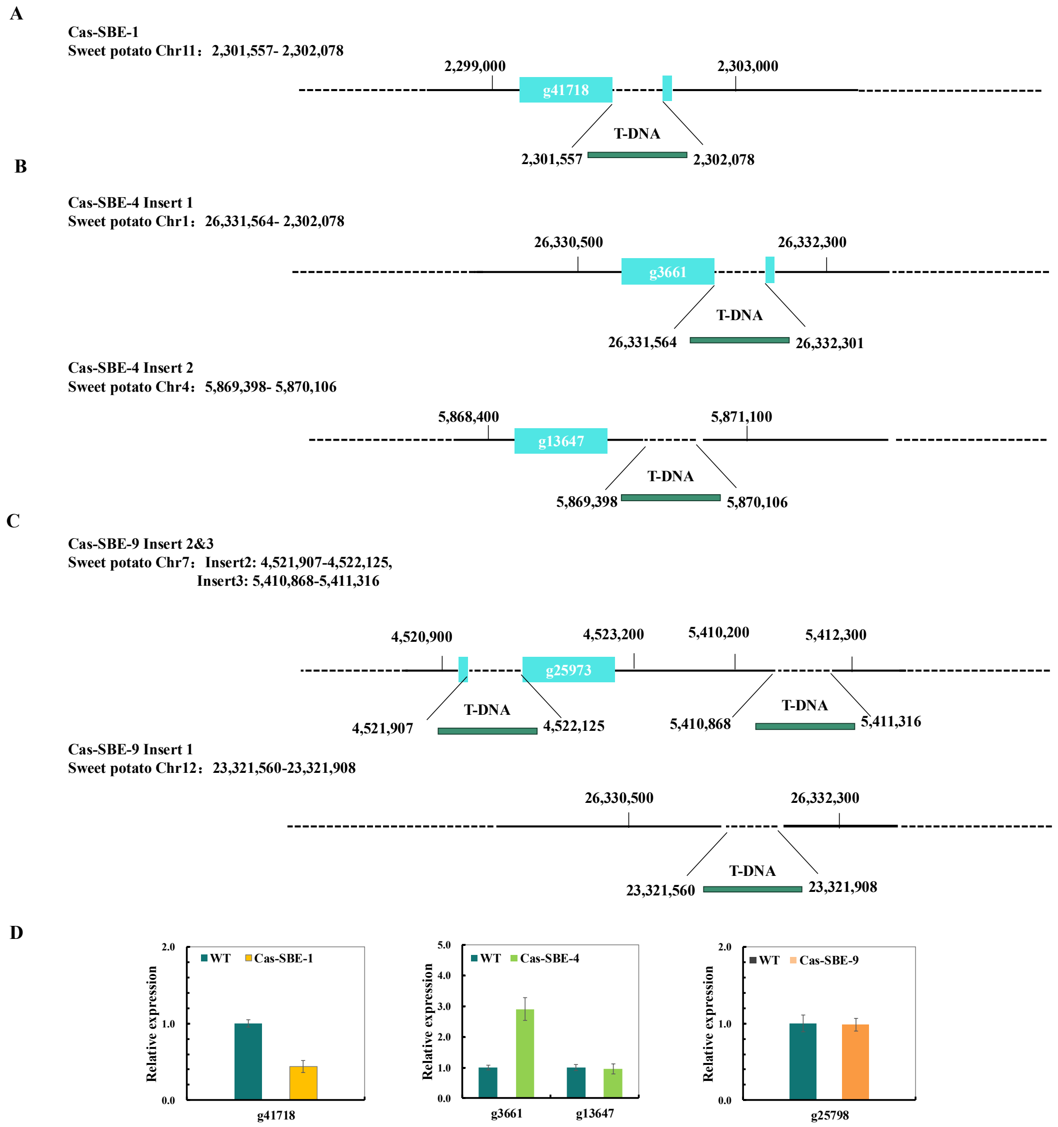

Figure S1. Identification of T-DNA insertion sites in CRISPR/Cas9-edited sweet potato lines. (A-C) Schematic diagrams of the insertional T-DNA integrations in the Cas-SBE-1 (A), Cas-SBE-4 (B), and Cas-SBE-9 (C) genomes. The numbers near the line of Chr. indicate the physical positions on these chromosomes. (D) Expression analysis of genes associated with T-DNA insertion sites.

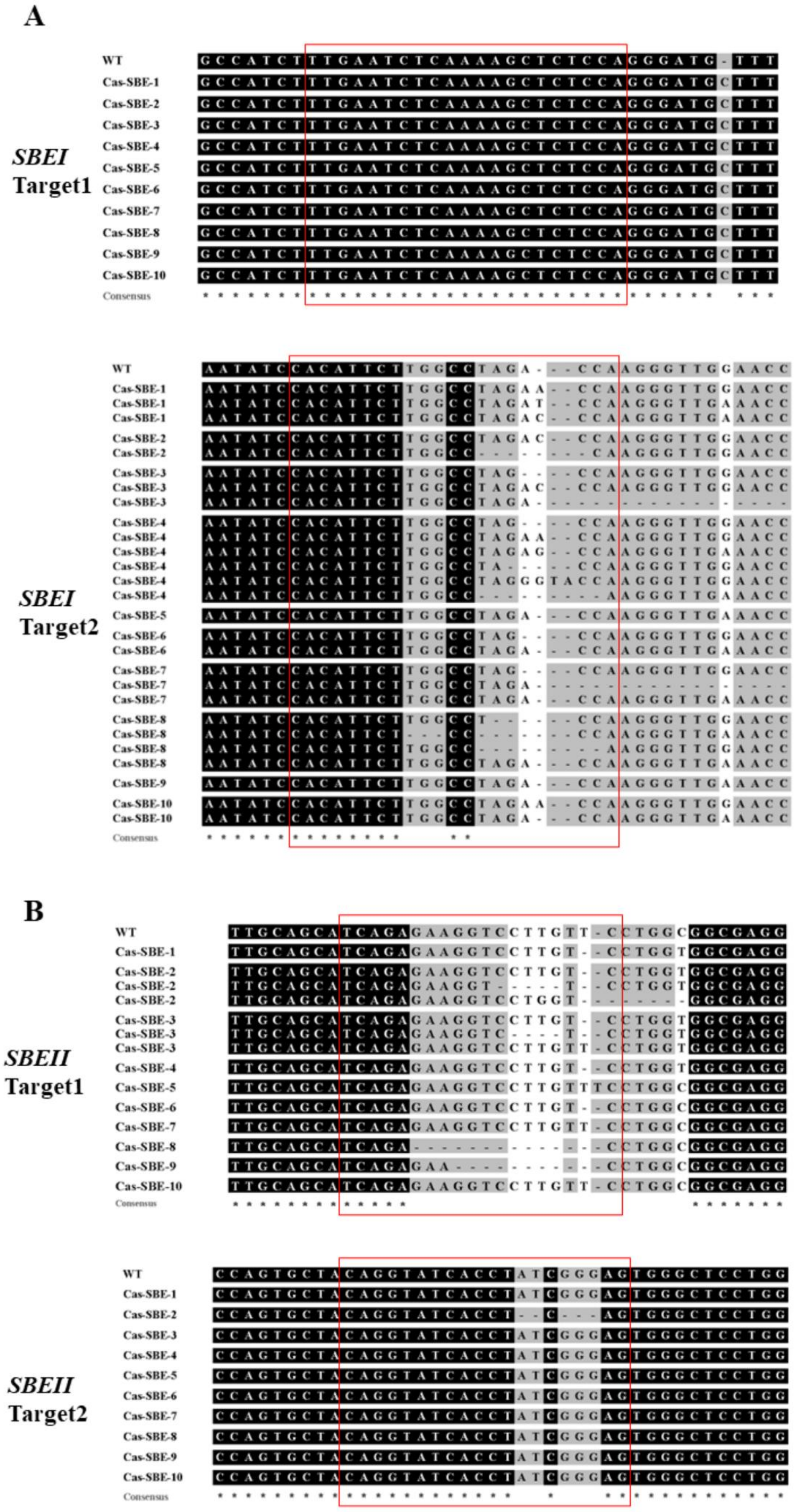
A
SBEI
Target1
WT
Cas-SBE-1
Cas-SBE-2
Cas-SBE-3
Cas-SBE-4
Cas-SBE-5
Cas-SBE-6
Cas-SBE-7
Cas-SBE-8
Cas-SBE-9
Cas-SBE-10
Consensus
SBEI
Target2
B
SBEII
Target1
SBEII
Target2

Figure S2. Sequence alignments of the four sgRNA target regions in SBEI and SBEII in wild-type and CRISPR/Cas9-edited sweet potato lines. (A) Alignments of SBEI Target 1 (top) and SBEI Target 2 (bottom). (B) Alignments of SBEII Target 1 (top) and SBEII Target 2 (bottom). For each target region, the wild-type (WT) sequence is aligned with the mutant haplotypes identified by Hi-TOM in the independent edited lines (Cas-SBE-1 to Cas-SBE-10); where a line carries more than one haplotype, each haplotype is shown on a separate row. Black and gray shading indicate columns with 100% and ≥75% sequence identity among all aligned sequences, respectively, and "-" denotes a gap (one nucleotide position). The Consensus row marks fully conserved positions with asterisks. Red boxes indicate the each sgRNA target.

Table S1 List of the primers used in this study

Primer name	Sequence 5’-3’	Purpose
SBEIFP	GGGCATTCCAGACAAGTGGA	qPCR
SBEIRP	CGCCACTACAAGATGCAAGC	qPCR
SBEIIFP	GACGGCAGAACACCAGTACA	qPCR
SBEIIRP	TCTGCCAAAGCCTCCAAACA	qPCR
g41718FP	TCAGTGCTTCCTCAGTTGCG	qPCR
g41718RP	AGAGTCCATAACCCAAAGCGG	qPCR
g3661FP	AAACCCGGCACTCATCGATT	qPCR
g3661RP	ATGTCCTGCTTCGCCCATTT	qPCR
g13647FP	AAGGCTGAGACGGATGCTTC	qPCR
g13647RP	ACCTTCCTCCAACTCCCCTT	qPCR
g25973FP	TGAAATTTGGAGGGTGCGGT	qPCR
g25973RP	CGTGGCAAGCACAGACAATC	qPCR
ActinFP	CTGGTGTTATGGTTGGGATGG	qPCR
ActinRP	GGGGTGCCTCGGTAAGAAG	qPCR

Table S2**.** Hi-TOM quantification of mutation spectra at the four sgRNA target sites in the Cas-SBE lines.

**Target site**	**Line**	**Variation type**	**Reads number**	**Reads ratio (%)**
	Cas-SBE-1	1I	10238	70.23
		WT	4015	28.54
	Cas-SBE-2	WT	7513	53.26
SBEI-Target1		1I	5372	38.90
	Cas-SBE-3	1I	7866	58.19
		WT	5038	44.82
	Cas-SBE-4	1I	9114	72.82

		WT	3125	23.52
	Cas-SBE-5	1I	38173	64.90
		WT	15859	28.88
	Cas-SBE-6	1I	39009	63.20
		WT	23208	36.16
	Cas-SBE-7	1I	42965	67.80
		WT	17414	28.45
	Cas-SBE-8	1I	43307	69.05
		WT	16281	26.21
	Cas-SBE-9	1I	44638	69.16
		WT	17075	26.46
	Cas-SBE-10	1I	45033	71.19
		WT	16832	26.39
SBEI-Target2	Cas-SBE-1	1I	22028	34.13
		WT	20098	31.14
		1I, SNP	10956	16.98
		1I, SNP	10722	16.61
	Cas-SBE-2	WT	23585	38.38
		1I	13221	21.51
		5D	12898	20.99
	Cas-SBE-3	WT	33408	52.67
		1D	11235	17.39
		1I	11017	17.05
		Large_Indel	8009	12.39
	Cas-SBE-4	6D, SNP	12376	18.87
		1D	11109	16.94
		1I, SNP	11071	16.88
		2D	10787	16.45
		1I	10774	16.43
		3I	9456	14.42
	Cas-SBE-5	WT	39288	63.90
		SNP	17648	28.70
	Cas-SBE-6	WT	25057	38.31
		SNP	24251	37.08
		1D	14103	21.56
	Cas-SBE-7	WT	20526	32.39
		35D,5D	14709	23.21
		1D	13940	22.00
		SNP	9070	14.31

	Cas-SBE-8	WT	22462	35.48
		4D, 4D, SNP	11398	18.00
		3D	10864	17.16
		SNP	9320	14.72
		6D	8530	13.47
	Cas-SBE-9	WT	31064	48.79
		SNP	28291	45.30
	Cas-SBE-10	WT	31645	48.56
		SNP	20570	31.57
		1I	12134	18.62
SBEII-Target1	Cas-SBE-1	WT	8958	78.29
		1D, SNP	1864	17.93
	Cas-SBE-2	7D, SNP	3240	22.64
		6D, SNP	2726	19.05
		1D, SNP	2576	18.00
		SNP	1761	12.31
	Cas-SBE-3	1D, SNP	4886	33.81
		5D, SNP	2921	20.21
		SNP	2273	15.73
	Cas-SBE-4	WT	10115	77.82
		1D, SNP	2007	16.45
	Cas-SBE-5	WT	68784	83.66
		1I	12183	14.60
	Cas-SBE-6	WT	63176	81.29
		1D	11742	13.13
	Cas-SBE-7	WT	62707	97.44
	Cas-SBE-8	WT	57331	79.95
		13D	9745	15.88
	Cas-SBE-9	9D	45028	69.18
		WT	20062	30.82
	Cas-SBE-10	WT	63671	96.63
SBEII-Target2	Cas-SBE-1	WT	87182	98.60
	Cas-SBE-2	WT	38811	79.54
		2D,3D	7698	14.04
	Cas-SBE-3	WT	40812	96.80
	Cas-SBE-4	WT	63300	97.21
	Cas-SBE-5	WT	40514	96.94
	Cas-SBE-6	WT	40102	97.61
	Cas-SBE-7	WT	45911	95.84

	Cas-SBE-8	WT	41894	96.78
	Cas-SBE-9	WT	63096	97.81
	Cas-SBE-10	WT	41558	95.96

WT, wild-type allele; SNP, single-nucleotide substitution; nI and nD, insertion and deletion of n bp, respectively (e.g., 1I, 35D); Large_Indel, large insertion/deletion; combined notations (e.g., “1I, SNP”) indicate multiple changes carried by the same allele. Reads ratio, percentage of reads supporting each allele among all reads at the target site.

Table S3. Thermal properties of *SBE*-mutated sweet potato determined by a differential scanning calorimeter.

Lines	To (℃)	Tp (℃)	Tc (℃)	△H (J/g)
WT	59.71±0.23	67.11±0.31	84.01±0.16	12.63±0.38
Cas-SBE-1	60.37±0.12	66.33±0.19	73.33±0.21	7.26±0.51
Cas-SBE-4	63.74±0.18	69.03±0.40	86.34±0.25	11.85±0.14
Cas-SBE-9	61.42±0.26	66.64±0.20	86.92±0.62	12.27±0.56
